# Supplementary material for: Evolution of kdr haplotypes in worldwide populations of Aedes aegypti: Independent origins of the F1534C kdr mutation
Source: PLoS Negl Trop Dis. 2020 Apr 16;14(4):e0008219. doi: 10.1371/journal.pntd.0008219 (PMC7188295; doi:10.1371/journal.pntd.0008219)
Supplement: S1 Table — (PDF) [file pntd.0008219.s001.pdf]

Supplementary Table S1

| Region | Country | City | Sample names | Year of Collection | number of mosquitos | Latitude | Longitude | Barcode Name | Barcode sequence | Bases | >=Q20 Bases | Reads | Average Length (bp) |
|---|---|---|---|---|---|---|---|---|---|---|---|---|---|
| Central America | Mexico | Amacuzac | Amacuzac | 2014 | 32 | 18.600 | -99.370 | IonXpress_092 | CTAGGAACCGC | 71,417,388 | 53,333,584 | 198,111 | 360 |
| South America | Brazil | Aracaju | Aracaju02 | 2002 | 30 | -10.947 | -37.073 | IonXpress_001 | CTAAGGTAAC | 577,747 | 479,349 | 2,033 | 284 |
| South America | Brazil | Aracaju | Aracaju06 | 2006 | 30 | -10.947 | -37.073 | IonXpress_002 | TAAGGAGAAC | 11,391,756 | 8,479,525 | 31,341 | 363 |
| South America | Brazil | Aracaju | Aracaju12 | 2001 | 30 | -10.947 | -37.073 | IonXpress_003 | AAGAGGATTC | 824,965 | 727,976 | 2,359 | 350 |
| South America | Brazil | Araçatuba | Aracatuba04 | 2004 | 30 | -21.203 | -50.454 | IonXpress_004 | TACCAAGATC | 10,366,382 | 7,855,338 | 28,349 | 366 |
| South America | Brazil | Araçatuba | Aracatuba07 | 2007 | 30 | -21.203 | -50.454 | IonXpress_005 | CAGAAGGAAC | 7,238,572 | 5,576,287 | 20,433 | 354 |
| South America | Brazil | Araçatuba | Aracatuba14 | 2014 | 30 | -21.203 | -50.454 | IonXpress_006 | CTGCAAGTTC | 252,440 | 220,996 | 826 | 306 |
| South America | Brazil | Araçatuba | Aracatuba14 | 2014 | 30 | -21.203 | -50.454 | IonXpress_067 | TTCCTACCAGTC | 35,636,915 | 27,639,240 | 91,386 | 390 |
| South America | Brazil | Araguaina | Araguaina06 | 2006 | 30 | -7.193 | -48.205 | IonXpress_007 | TTCGTGATTC | 8,461,146 | 5,994,157 | 27,557 | 307 |
| South America | Brazil | Araguaina | Araguaina12 | 2012 | 30 | -7.193 | -48.205 | IonXpress_008 | TTCCGATAAC | 36,575,647 | 26,280,539 | 95,497 | 383 |
| South America | Brazil | Araguatins | Araguatina12 | 2012 | 30 | -5.648 | -48.119 | IonXpress_059 | TCCTTGATGTTC | 25,060,184 | 19,616,327 | 64,054 | 391 |
| Asia | Thailand | Bangkok | Bangkok | 2013 | 32 | 13.756 | 100.502 | IonXpress_065 | TCCTGGCACATC | 20,960,710 | 15,848,175 | 54,914 | 382 |
| South America | Brazil | Belém | Belem10 | 2010 | 30 | -1.456 | -48.490 | IonXpress_009 | TGAGCGGAAC | 3,505,003 | 3,100,535 | 9,156 | 383 |
| Africa | Guinea Bissau | Bijagos | GBissau | 2009 | 23 | 11.804 | -15.180 | IonXpress_086 | CTTGGTTATTC | 15,157,265 | 11,247,335 | 41,393 | 366 |
| South America | Brazil | Boa Vista | BVista10 | 2010 | 30 | 2.492 | -60.403 | IonXpress_011 | TCCTCGAATC | 127,019 | 110,445 | 375 | 339 |
| South America | Brazil | Cabo Frio | CFrio02 | 2002 | 30 | -22.683 | -42.037 | IonXpress_032 | TCTTACACAC | 1,323,746 | 1,061,165 | 3,510 | 377 |
| South America | Brazil | Cabo Frio | CFrio08 | 2008 | 30 | -22.683 | -42.037 | IonXpress_033 | TTCTCATTGAAC | 5,721,893 | 4,963,914 | 14,694 | 389 |
| Oceania | Australia | Cairms | Australia | 2013 | 32 | -16.557 | -145.464 | IonXpress_084 | CTTCCATAAC | 3,535,300 | 2,770,728 | 9,106 | 388 |
| South America | Colombia | Cali | Cali | 2013 | 32 | 3.452 | -76.532 | IonXpress_088 | CCGAACACTTC | 39,302,839 | 29,317,072 | 105,655 | 372 |
| North America | USA | California | California | 2013 | 29 | 36.778 | -119.418 | IonXpress_081 | CCTGCCATTCGC | 24,017,551 | 18,711,583 | 61,532 | 390 |
| South America | Brazil | Campinas | Campinas04 | 2004 | 30 | -22.933 | -47.074 | IonXpress_061 | TCACTCGGATC | 11,142,401 | 8,782,606 | 29,563 | 377 |
| South America | Brazil | Campinas | Campinas14 | 2014 | 30 | -22.933 | -47.074 | IonXpress_068 | TCAAGAAGTTC | 8,291,379 | 6,452,500 | 21,396 | 388 |
| South America | Brazil | Campo Grande | CGrande10 | 2010 | 30 | -20.281 | 54.371 | IonXpress_012 | TAGGTGGTTC | 582,635 | 514,687 | 1,515 | 385 |
| South America | Brazil | Campos dos Goytacaze | Campos03 | 2003 | 30 | -21.759 | -41.327 | IonXpress_058 | TCCTAGAACAC | 6,636,947 | 5,111,885 | 17,722 | 375 |
| Asia | Philippines | Cebu | Cbu | 2013 | 32 | 12.880 | 121.774 | IonXpress_064 | CTGAGTTCCGAC | 20,805,930 | 16,141,621 | 53,439 | 389 |
| Central America | Dominica | Dominica | Dominica | 2009 | 32 | 15.415 | -61.371 | IonXpress_063 | CCTTAGAGTTC | 17,805,671 | 13,883,748 | 46,953 | 379 |
| South America | Brazil | Duque de Caxias | DCaxias01 | 2001 | 30 | -22.786 | -43.305 | IonXpress_013 | TCTAACGGAC | 21,475,625 | 16,196,894 | 56,419 | 381 |
| South America | Brazil | Duque de Caxias | DCaxias10 | 2010 | 30 | -22.786 | -43.305 | IonXpress_014 | TTGGAGTGTC | 967,645 | 836,956 | 2,604 | 372 |
| South America | Brazil | Fortaleza | Fortaleza04 | 2004 | 30 | -3.436 | -38.314 | IonXpress_015 | TCTAGAGGTC | 101,511 | 90,629 | 282 | 360 |
| South America | Brazil | Foz do Iguaçu | FIguacu06 | 2006 | 30 | -25.306 | -54.357 | IonXpress_016 | TCTGGATGAC | 584,365 | 388,565 | 1,684 | 347 |
| South America | Brazil | Goiânia | Goiânia10 | 2010 | 30 | -16.687 | -49.264 | IonXpress_018 | AGGCAATTGC | 4,975,093 | 4,169,077 | 13,959 | 356 |
| Africa | Senegal | Goudiri | Goudiry | 2012 | 30 | 14.185 | -12.716 | IonXpress_077 | CGAAGCGATTC | 24,362,523 | 19,057,940 | 64,130 | 380 |
| South America | Brazil | Governador Valadares | GValadares11 | 2011 | 30 | -18.855 | -41.956 | IonXpress_019 | TTAGTCGGAC | 3,921,064 | 3,353,823 | 10,443 | 375 |
| Central America | Haiti | Haiti | Haiti | 2010 | 30 | 18.971 | -72.285 | IonXpress_050 | CGGACAATGGC | 17,081,573 | 13,237,071 | 44,232 | 386 |
| Pacific | USA | Hawaii | Hawaii | 2009 | 25 | 19.894 | -155.582 | IonXpress_091 | CGGAAGGATGC | 19,567,463 | 15,034,721 | 53,149 | 368 |
| Central America | Mexico | Iguala | Iguala | 2012 | 32 | 18.345 | -99.540 | IonXpress_085 | CCAGCCTCAAC | 18,514,464 | 14,399,287 | 48,362 | 383 |
| South America | Brazil | Itacoatiara | Itacoatiara15 | 2015 | 30 | -3.335 | -58.554 | IonXpress_066 | CCGCAATCATC | 34,390,761 | 26,838,313 | 96,584 | 356 |
| South America | Brazil | Itaperuna | Itaperuna02 | 2002 | 30 | -21.238 | -41.898 | IonXpress_028 | ATCCGGAATC | 6,285,000 | 5,525,119 | 16,324 | 385 |
| Asia | Saudi Arabia | Jeddah | SArabia | 2012 | 32 | 21.486 | 39.192 | IonXpress_095 | CGGACAGATC | 62,550,861 | 47,872,422 | 160,586 | 390 |
| Africa | Uganda | Lunyo | Lunyo | 2013 | 31 | 0.072 | 32.462 | IonXpress_083 | CTAGGACATTC | 8,470,227 | 6,317,147 | 21,713 | 390 |
| South America | Brazil | Macapá | Macapá14 | 2014 | 30 | 0.036 | -51.071 | IonXpress_020 | CAGATCCATC | 4,463,093 | 3,945,396 | 11,593 | 385 |
| South America | Brazil | Maceió | Maceio09 | 2009 | 30 | -9.650 | -35.709 | IonXpress_021 | TCGCAATTAC | 4,816,274 | 4,185,606 | 12,541 | 384 |
| South America | Brazil | Manaus | Manaus09 | 2009 | 30 | -3.119 | -60.022 | IonXpress_022 | TTCGAGACGC | 2,375,399 | 1,986,854 | 6,803 | 349 |
| South America | Brazil | Marabá | Maraba10 | 2010 | 30 | -5.593 | -50.182 | IonXpress_023 | TGCCACGAAC | 9,314,920 | 8,223,810 | 24,158 | 386 |
| South America | Brazil | Marilia | Marilia14 | 2014 | 30 | -22.218 | -49.951 | IonXpress_069 | TTCAATTGGC | 22,063,645 | 17,105,151 | 57,343 | 385 |
| South America | Brazil | Marília | Marilia11 | 2011 | 30 | -22.218 | -49.951 | IonXpress_055 | TCCACCTCCTC | 3,370,549 | 2,590,706 | 10,788 | 312 |

Supplementary Table S1

| Region | Country | City | Sample names | Year of Collection | number of mosquitos | Latitude | Longitude | Barcode Name | Barcode sequence | Bases | >=Q20 Bases | Reads | Average Length (bp) |
|---|---|---|---|---|---|---|---|---|---|---|---|---|---|
| South America | Brazil | Marília | Marilia04 | 2004 | 30 | -22.218 | -49.951 | IonXpress_074 | CGATCGGTTC | 43,133,652 | 33,269,030 | 134,724 | 320 |
| Central America | Mexico | Mazatan | Mazatan | 2012 | 32 | 14.861 | -92.448 | IonXpress_090 | CTAACCACGGC | 21,247,678 | 16,451,733 | 56,465 | 376 |
| South America | Brazil | Montes Claros | MClaros06 | 2006 | 30 | -16.434 | -43.513 | IonXpress_024 | AACCTCATTC | 11,127,137 | 9,879,189 | 29,097 | 382 |
| South America | Brazil | Mossoró | Mossoro09 | 2009 | 30 | -5.184 | -37.348 | IonXpress_025 | CCTGAGATAC | 5,842,423 | 4,914,301 | 15,142 | 386 |
| South America | Brazil | Mossoró | Mossoro11 | 2011 | 30 | -5.184 | -37.348 | IonXpress_026 | TTACAACCTC | 4,806,993 | 4,095,171 | 12,379 | 388 |
| Africa | Kenya | Nairobi | Kenya | 2012 | 32 | -0.024 | 37.906 | IonXpress_096 | TTAAGCGGTC | 37,176,652 | 29,087,317 | 99,329 | 374 |
| North America | USA | New Orleans | NOrleans | 2012 | 30 | 29.951 | -90.072 | IonXpress_082 | TTGGCATCTC | 25,851,646 | 20,136,436 | 66,502 | 389 |
| Africa | Senegal | Ngari | Ngari | 2012 | 16 | 14.412 | -12.907 | IonXpress_080 | TCGAAGGCAGGC | 14,395,728 | 11,025,557 | 37,813 | 381 |
| South America | Brazil | Niterói | Niteroi01 | 2001 | 30 | -22.886 | -43.115 | IonXpress_027 | AACCATCCGC | 3,184,819 | 2,650,072 | 8,445 | 377 |
| South America | Brazil | Niterói | Niteroi01 | 2001 | 30 | -22.886 | -43.115 | IonXpress_057 | TCTGGCAACGGC | 22,634,192 | 17,691,125 | 58,592 | 386 |
| South America | Brazil | Nova Iguaçu | NIguaçu09 | 2009 | 30 | -22.756 | -43.461 | IonXpress_029 | TCGACCACTC | 2,137,617 | 1,791,237 | 5,677 | 377 |
| South America | Brazil | Nova Iguaçu | NIguacu03 | 2003 | 30 | -22.756 | -43.461 | IonXpress_062 | TTCCTGCTTCAC | 14,799,439 | 11,234,258 | 38,733 | 382 |
| South America | Brazil | Oiapoque | Oiapoque14 | 2014 | 30 | 3.845 | -51.833 | IonXpress_030 | CGAGGTTATC | 1,121,906 | 958,862 | 2,869 | 391 |
| South America | Brazil | Pacaraima | Pacaraima11 | 2011 | 30 | 4.479 | -61.147 | IonXpress_031 | TCCAAGCTGC | 777,472 | 675,959 | 2,053 | 379 |
| South America | Brazil | Palmas | Palmas05 | 2005 | 30 | -10.249 | -48.324 | IonXpress_034 | TCGCATCGTTC | 3,806,540 | 3,348,807 | 10,130 | 376 |
| South America | Brazil | Palmas | Palmas12 | 2012 | 30 | -10.249 | -48.324 | IonXpress_035 | TAAGCCATTGTC | 27,527,387 | 24,262,873 | 70,977 | 388 |
| South America | Brazil | Parnaíba | Parnaiba05 | 2005 | 30 | -2.906 | -41.773 | IonXpress_052 | CCGCATGGAAC | 1,912,701 | 1,496,744 | 4,959 | 386 |
| South America | Brazil | Parnamirim | Parnamirim09 | 2009 | 30 | -5.544 | -35.162 | IonXpress_036 | AAGGAATCGTC | 3,679,281 | 3,224,563 | 9,586 | 384 |
| South America | Brazil | Presidente Prudente | PPrudente14 | 2014 | 30 | -22.814 | -51.232 | IonXpress_070 | CCTACTGGTC | 30,533,913 | 23,202,800 | 83,966 | 364 |
| Central America | USA | Puerto Rico | PuertoRico | 2014 | 31 | 18.220 | -66.589 | IonXpress_079 | CCTGGTTGTC | 27,806,860 | 20,947,234 | 71,096 | 391 |
| South America | Brazil | Ribeirão Preto | RibPreto09 | 2009 | 30 | -21.178 | -47.813 | IonXpress_056 | CAGCATTAATTC | 21,982,714 | 17,543,867 | 56,809 | 387 |
| South America | Brazil | Ribeirão Preto | RibPreto14 | 2014 | 30 | -21.178 | -47.813 | IonXpress_071 | TGAGGCTCCGAC | 14,346,840 | 11,519,780 | 38,451 | 373 |
| South America | Brazil | Ribeirão Preto | RibPreto11 | 2011 | 30 | -21.178 | -47.813 | IonXpress_073 | TCTGCCTGTC | 32,907,078 | 25,062,610 | 85,489 | 385 |
| South America | Brazil | Rio Branco | RBranco05 | 2005 | 30 | -9.975 | -68.429 | IonXpress_037 | CTTGAGAATGTC | 2,370,424 | 2,047,850 | 6,389 | 371 |
| South America | Brazil | Rio Branco | RBranco11 | 2011 | 30 | -9.975 | -68.429 | IonXpress_038 | TGGAGGACGGAC | 1,850,821 | 1,621,579 | 4,840 | 382 |
| South America | Brazil | Salvador | Salvador08 | 2008 | 30 | -12.584 | -38.306 | IonXpress_039 | TAACAATCGGC | 1,085 | 671 | 5 | 217 |
| South America | Brazil | Salvador | Salvador11 | 2011 | 30 | -12.584 | -38.306 | IonXpress_040 | CTGACATAATC | 285 | 254 | 2 | 143 |
| South America | Brazil | Santa Bárbara | SBarbara08 | 2008 | 30 | -28.385 | -53.258 | IonXpress_049 | TCCTAACATAAC | 24,901,187 | 17,877,093 | 65,955 | 378 |
| South America | Brazil | Santa Rosa | SRosa06 | 2006 | 30 | -21.178 | -47.813 | IonXpress_041 | TTCCACTTCGC | 178 | 178 | 1 | 178 |
| South America | Brazil | Santa Rosa | SRosa11 | 2011 | 30 | -21.178 | -47.813 | IonXpress_042 | AGCACGAATC | 272 | 262 | 1 | 272 |
| South America | Brazil | Santarém | Santarem10 | 2010 | 30 | -2.272 | -54.423 | IonXpress_043 | CTTGACACCGC | 1,639 | 1,567 | 6 | 273 |
| South America | Brazil | Santos | Santos11 | 2011 | 30 | -23.554 | -46.215 | IonXpress_044 | TTGGAGGCCAGC | 777 | 543 | 3 | 259 |
| South America | Brazil | Santos | Santos14 | 2014 | 30 | -23.554 | -46.215 | IonXpress_045 | TGGAGCTTCCTC | 1,864 | 1,614 | 6 | 311 |
| South America | Brazil | São Gonçalo | SGonçalo01 | 2001 | 30 | -22.494 | -43.350 | IonXpress_046 | TCAGTCCGAAC | 396 | 389 | 1 | 396 |
| South America | Brazil | São Gonçalo | SGonçalo08 | 2008 | 30 | -22.494 | -43.350 | IonXpress_047 | TAAGGCAACCAC | 305 | 290 | 1 | 305 |
| South America | Brazil | São José do Rio Preto | SJRPreto08 | 2008 | 30 | -20.812 | -49.376 | IonXpress_051 | TTGAGCCTATTC | 12,410,461 | 9,468,898 | 32,924 | 377 |
| South America | Brazil | São José do Rio Preto | SJRPreto14 | 2014 | 30 | -20.812 | -49.376 | IonXpress_072 | CGAAGGCCACAC | 30,835,395 | 24,264,275 | 82,675 | 373 |
| Africa | Senegal | Sedhiou | Sedhiou | 2012 | 31 | 12.884 | -15.594 | IonXpress_076 | CGGAAGAACCTC | 52,516,637 | 40,351,725 | 140,448 | 374 |
| Pacific | Tahiti | Tahiti | Tahiti | 2010 | 30 | -17.651 | -149.426 | IonXpress_078 | CAGCCAATTCTC | 12,296 | 10,267 | 37 | 332 |
| Oceania | Australia | Townsville | Townsville | 2009 | 18 | -19.259 | 146.817 | IonXpress_093 | CTTGTCCAATC | 29,284,159 | 19,683,499 | 77,395 | 378 |
| South America | Brazil | Tubiacanga | Tubiacanga15 | 2015 | 30 | -22.475 | -43.133 | IonXpress_048 | TTCTAAGAGAC | 0 | 0 | 0 | N/A |
| North America | USA | Tuson | Tucson | 2012 | 32 | 32.223 | -110.975 | IonXpress_087 | TTGGCTGGAC | 29,508,573 | 22,577,970 | 77,411 | 381 |
| South America | Brazil | Urca | Urca15 | 2015 | 30 | -22.954 | -43.168 | IonXpress_060 | TCTAGCTCTTC | 16,320,729 | 12,346,797 | 43,384 | 376 |
| South America | Brazil | Vila Velha | VVelha06 | 2006 | 30 | -20.348 | -40.295 | IonXpress_075 | TCAGGAATAC | 38,614,966 | 29,879,255 | 139,591 | 277 |
| Africa | Cameroon | Yaounde | Yaounde | 2014 | 32 | 3.848 | 11.502 | IonXpress_094 | TCCGACAAGC | 30,798,059 | 22,851,904 | 80,907 | 381 |
| South America | Venezuela | Zulia | Zulia | 2004 | 32 | 10.291 | -72.141 | IonXpress_089 | TCCTGAATCTC | 24,388,957 | 17,873,213 | 63,764 | 382 |
| | | | 92 samples | | | | | Total | | 1,322,931,619 | 1,020,906,422 | 3,561,595 | 360.4505495 |
