# Supplementary material for: Evolution of kdr haplotypes in worldwide populations of Aedes aegypti: Independent origins of the F1534C kdr mutation
Source: PLoS Negl Trop Dis. 2020 Apr 16;14(4):e0008219. doi: 10.1371/journal.pntd.0008219 (PMC7188295; doi:10.1371/journal.pntd.0008219)
Supplement: S2 Table — (PDF) [file pntd.0008219.s002.pdf]

Region	Country	City	Year of Collection	state	reads	bases	max	min	N50	N90
Central America	Mexico	Amacuzac	2014	Raw	188,683	55,230,051	614	25	376	211
				Filtered	87,225	33,660,386	400	350	393	360
South America	Brazil	Aracaju	2002	Raw	1,849	488,146	432	25	376	179
				Filtered	757	287,756	400	350	377	375
South America	Brazil	Aracaju	2006	Raw	29,666	8,859,440	493	25	372	235
				Filtered	13,217	5,078,424	400	350	392	359
South America	Brazil	Aracaju	2001	Raw	2,343	758,897	414	25	378	253
				Filtered	1613	619,754	400	350	390	376
South America	Brazil	Araçatuba	2004	Raw	27,033	8,221,255	486	25	376	249
				Filtered	14,482	5,537,249	400	350	379	365
South America	Brazil	Araçatuba	2007	Raw	19,436	5,822,772	483	25	377	209
				Filtered	10540	4,073,210	400	350	394	373
South America	Brazil	Araçatuba	2014	Raw	813	227,553	405	25	377	164
				Filtered	418	160,943	400	350	392	376
South America	Brazil	Araçatuba	2014	Raw	87,690	28,511,099	494	25	395	289
				Filtered	60637	23,685,861	400	350	395	376
South America	Brazil	Araguaina	2006	Raw	25,341	6,081,028	462	25	356	147
				Filtered	8,676	3,259,358	400	350	376	360
South America	Brazil	Araguaina	2012	Raw	87,834	26,802,406	522	25	395	253
				Filtered	47478	18,595,120	400	350	395	382
South America	Brazil	Araguatins	2012	Raw	60,747	20,249,131	478	25	395	316
				Filtered	44,282	17,392,187	400	350	395	388
Oceania	Australia	Cairms	2013	Raw	8,703	2,927,649	612	25	395	285
				Filtered	6140	2,401,526	400	350	395	375
Asia	Thailand	Bangkok	2013	Raw	52,101	16,354,915	467	25	376	265
				Filtered	33,566	12,884,369	400	350	386	374
South America	Brazil	Boa Vista	2010	Raw	369	114,185	402	28	377	254
				Filtered	242	91,478	398	357	377	375
South America	Brazil	Belém	2010	Raw	9,077	3,210,736	443	25	377	339
				Filtered	7,403	2,832,505	400	350	378	376
South America	Brazil	Cabo Frio	2002	Raw	3,408	1,078,160	486	25	378	266
				Filtered	1986	759,612	400	350	383	363
South America	Brazil	Cabo Frio	2008	Raw	14,573	5,136,123	464	25	392	334
				Filtered	11,396	4,419,017	400	350	392	375
South America	Brazil	Campo Grande	2010	Raw	1,506	533,518	405	28	392	347
				Filtered	1224	475,122	400	350	392	375
South America	Colombia	Cali	2013	Raw	100,540	30,207,293	575	25	377	224
				Filtered	53,728	20,715,510	400	350	393	361
North America	USA	California	2013	Raw	59,420	19,427,490	557	25	393	281
				Filtered	38697	15,050,029	400	350	395	363
South America	Brazil	Campinas	2004	Raw	28,968	9,316,306	487	25	376	255
				Filtered	17,405	6,642,211	400	350	380	361
South America	Brazil	Campinas	2014	Raw	20,554	6,722,909	449	25	390	274
				Filtered	13742	5,305,989	400	350	393	374
South America	Brazil	Campos dos Goytacazes	2003	Raw	16,998	5,384,658	432	25	376	250
				Filtered	9,994	3,817,569	400	350	390	359
South America	Brazil	Duque de Caxias	2001	Raw	53,220	16,828,935	551	25	392	247
				Filtered	31901	12,348,225	400	350	394	366
South America	Brazil	Duque de Caxias	2010	Raw	2,584	854,027	458	25	392	258
				Filtered	1,774	686,900	400	350	392	376
Central America	Dominica	Dominica	2009	Raw	44,990	14,436,915	490	25	392	255
				Filtered	27580	10,720,734	400	350	395	374
South America	Brazil	Foz do Iguaçu	2006	Raw	1,543	398,340	436	25	352	171
				Filtered	549	206,456	397	350	376	353
South America	Brazil	Fortaleza	2004	Raw	279	94,439	398	26	377	268
				Filtered	201	77,068	398	355	379	376
Africa	Guinea Bissau	Bijagos	2009	Raw	39,050	11,699,360	572	25	363	223
				Filtered	17,020	6,414,238	400	350	376	363
South America	Brazil	Governador Valadares	2011	Raw	10,346	3,406,514	499	25	381	281
				Filtered	6992	2,689,515	400	350	392	373
South America	Brazil	Goiânia	2010	Raw	13,669	4,228,156	471	25	377	240
				Filtered	7,660	2,936,609	400	350	387	372
Africa	Senegal	Goudiri	2012	Raw	62,510	20,116,937	497	25	376	255
				Filtered	34318	13,222,039	400	350	393	361
Central America	Haiti	Haiti	2010	Raw	42,790	13,902,753	493	25	377	262
				Filtered	26,556	10,198,443	400	350	392	361
Pacific	USA	Hawaii	2009	Raw	51,080	15,774,749	618	25	376	241
				Filtered	27025	10,397,777	400	350	392	361
Central America	Mexico	Iguala	2012	Raw	46,874	15,027,017	486	25	383	257
				Filtered	28,797	11,093,705	400	350	392	363
South America	Brazil	Itacoatiara	2015	Raw	92,743	27,879,052	539	25	376	219
				Filtered	53295	20,496,747	400	350	383	375
South America	Brazil	Itaperuna	2002	Raw	16,230	5,770,717	482	25	391	335
				Filtered	13,032	5,020,949	400	350	392	376
Africa	Kenya	Nairobi	2012	Raw	94,195	30,298,276	540	25	395	255
				Filtered	58555	22,898,528	400	350	395	376
Africa	Uganda	Lunyo	2013	Raw	20,598	6,617,714	495	25	394	255
				Filtered	11,748	4,589,712	400	350	395	376
South America	Brazil	Montes Claros	2006	Raw	28,941	10,339,698	461	25	392	340
				Filtered	23355	9,039,976	400	350	392	376
South America	Brazil	Macapá	2014	Raw	11,538	4,092,558	472	25	377	339
				Filtered	9,370	3,588,940	400	350	378	376
South America	Brazil	Maceió	2009	Raw	12,404	4,327,191	468	25	378	322
				Filtered	9688	3,717,280	400	350	380	376
South America	Brazil	Manaus	2009	Raw	6,729	2,017,006	503	25	376	228
				Filtered	3,329	1,264,881	400	350	377	374
South America	Brazil	Marabá	2010	Raw	23,976	8,467,556	465	25	392	342
				Filtered	19169	7,411,325	400	350	393	376
South America	Brazil	Marília	2004	Raw	126,803	34,402,116	625	25	392	188
				Filtered	47,009	18,453,678	400	350	395	388
South America	Brazil	Marília	2011	Raw	10,213	2,702,613	466	25	342	187
				Filtered	3448	1,309,476	400	350	376	374

Region	Country	City	Year of Collection	state	reads	bases	max	min	N50	N90
South America	Brazil	Marilia	2014	Raw	54,378	17,825,170	619	25	392	274
				Filtered	36,745	14,246,442	400	350	395	374
Central America	Mexico	Mazatan	2012	Raw	54,124	17,181,684	511	25	376	256
				Filtered	32799	12,576,567	400	350	390	373
South America	Brazil	Mossoró	2009	Raw	14,963	5,065,435	518	25	380	293
				Filtered	10,453	4,021,936	400	350	392	374
South America	Brazil	Mossoró	2011	Raw	12,240	4,222,868	473	25	388	316
				Filtered	9088	3,500,762	400	350	392	375
South America	Brazil	Nova Iguaçu	2003	Raw	36,947	11,739,494	484	25	376	252
				Filtered	21,407	8,206,936	400	350	392	359
South America	Brazil	Nova Iguaçu	2009	Raw	5,631	1,831,514	492	25	390	271
				Filtered	3635	1,398,467	400	350	392	364
North America	USA	New Orleans	2012	Raw	64,018	20,760,049	532	25	394	284
				Filtered	43,039	16,801,836	400	350	395	376
Africa	Senegal	Ngari	2012	Raw	36,086	11,529,403	589	25	376	263
				Filtered	17518	6,715,008	400	350	377	374
South America	Brazil	Niterói	2001	Raw	56,165	18,590,191	484	25	377	273
				Filtered	37,446	14,377,199	400	350	392	366
South America	Brazil	Niterói	2001	Raw	8,296	2,713,207	516	25	377	276
				Filtered	5622	2,145,195	400	350	378	374
South America	Brazil	Oiapoque	2014	Raw	2,842	990,015	411	26	392	326
				Filtered	2,214	852,788	400	350	392	361
South America	Brazil	Presidente Prudente	2014	Raw	79,282	23,950,807	520	25	392	243
				Filtered	42940	16,684,656	400	350	395	375
South America	Brazil	Pacaraima	2011	Raw	2,029	693,116	469	25	377	315
				Filtered	1,545	591,649	400	350	378	376
South America	Brazil	Palmas	2005	Raw	10,071	3,489,944	515	25	377	321
				Filtered	7827	2,984,763	400	350	377	375
South America	Brazil	Palmas	2012	Raw	70,550	24,895,025	499	25	392	344
				Filtered	56,652	21,903,287	400	350	393	376
Asia	Philippines	Cebu	2013	Raw	52,141	17,110,690	475	25	384	258
				Filtered	32109	12,260,523	400	350	392	353
South America	Brazil	Parnaíba	2005	Raw	4,740	1,574,260	463	25	395	303
				Filtered	3,353	1,313,584	400	350	395	380
South America	Brazil	Parnamirim	2009	Raw	9,520	3,352,751	495	25	386	324
				Filtered	7503	2,889,418	400	350	392	376
Central America	USA	Puerto Rico	2014	Raw	67,241	21,590,324	488	25	392	260
				Filtered	41,099	15,849,319	400	350	392	354
South America	Brazil	Rio Branco	2005	Raw	6,317	2,120,566	461	25	376	288
				Filtered	4488	1,710,774	400	350	377	374
South America	Brazil	Rio Branco	2011	Raw	4,789	1,654,602	480	25	377	331
				Filtered	3,747	1,434,464	400	350	378	376
South America	Brazil	Ribeirão Preto	2009	Raw	55,668	18,571,274	471	25	390	280
				Filtered	37822	14,581,791	400	350	392	372
South America	Brazil	Ribeirão Preto	2011	Raw	81,450	25,910,521	505	25	392	256
				Filtered	51,435	19,924,337	400	350	394	372
South America	Brazil	Ribeirão Preto	2014	Raw	37,455	12,039,723	488	25	392	253
				Filtered	23516	9,125,017	400	350	394	375
Asia	Saudi Arabia	Jeddah	2012	Raw	155,156	49,740,298	547	25	391	261
				Filtered	101,072	39,022,118	400	350	394	374
South America	Brazil	Santa Bárbara	2008	Raw	62,108	18,450,273	485	25	376	225
				Filtered	31501	12,067,129	400	350	392	353
South America	Brazil	São José do Rio Preto	2008	Raw	31,673	9,889,734	482	25	376	252
				Filtered	18,468	7,047,106	400	350	379	362
South America	Brazil	São José do Rio Preto	2014	Raw	79,858	25,465,494	525	25	392	252
				Filtered	48814	18,938,634	400	350	395	374
Africa	Senegal	Sedhiou	2012	Raw	135,062	42,206,979	545	25	376	253
				Filtered	73,220	28,154,236	400	350	392	373
North America	USA	Tuson	2012	Raw	73,610	23,500,152	618	25	376	258
				Filtered	45747	17,508,909	400	350	386	371
Pacific	Tahiti	Tahiti	2010	Raw	36	10,802	397	48	389	175
				Filtered	20	7,732	397	351	395	367
Oceania	Australia	Townsville	2009	Raw	71,107	19,315,928	516	25	387	199
				Filtered	28031	10,914,928	400	350	395	371
South America	Brazil	Urca	2015	Raw	41,861	12,982,431	468	25	376	243
				Filtered	23,353	8,884,760	400	350	377	358
South America	Brazil	Vila Velha	2006	Raw	132,119	30,904,556	512	25	321	157
				Filtered	36181	14,216,705	400	350	395	391
Africa	Cameroon	Yaounde	2014	Raw	77,233	23,860,238	465	25	375	253
				Filtered	36,958	14,093,413	400	350	376	373
South America	Venezuela	Zulia	2004	Raw	59,520	18,271,759	542	25	392	244
				Filtered	33730	13,036,133	400	350	394	361
