# Supplementary material for: Evolution of kdr haplotypes in worldwide populations of Aedes aegypti: Independent origins of the F1534C kdr mutation
Source: PLoS Negl Trop Dis. 2020 Apr 16;14(4):e0008219. doi: 10.1371/journal.pntd.0008219 (PMC7188295; doi:10.1371/journal.pntd.0008219)
Supplement: S3 Table — (PDF) [file pntd.0008219.s003.pdf]

Supplementary Table S4

IIS6 haplotype frequencies

Populations*	2S6_B_00	2S6_A_01	2S6_A_02	2S6_A_03	2S6_B_04	2S6_B_05	2S6_B_06	2S6_B_07	2S6_B_08	2S6_B_09	2S6_B_10	2S6_B_11	2S6_B_12	2S6_B_13	2S6_B_14	2S6_B_15	2S6_B_16	2S6_B_17	2S6_B_18	2S6_A_19	2S6_B_20	2S6_B_21	2S6_A_22	2S6_B_23	2S6_B_24	2S6_B_25
AU_Cairns_13	0.0618102	0	0	0.395143	0.543046	0	0	0	0	0	0	0	0	0	0	0	0	0	0	0	0	0	0	0	0	0
AU_Townsville_09	0.216049	0	0	0.395944	0.388007	0	0	0	0	0	0	0	0	0	0	0	0	0	0	0	0	0	0	0	0	0
BR_Aracaju_02	0.825714	0	0.102857	0.0714286	0	0	0	0	0	0	0	0	0	0	0	0	0	0	0	0	0	0	0	0	0	0
BR_Aracaju_06	0.361282	0	0.0686395	0.463625	0.106453	0	0	0	0	0	0	0	0	0	0	0	0	0	0	0	0	0	0	0	0	0
BR_Aracaju_12	0.525698	0.143906	0.330396	0	0	0	0	0	0	0	0	0	0	0	0	0	0	0	0	0	0	0	0	0	0	0
BR_Aracatuba_04	0.807813	0.192187	0	0	0	0	0	0	0	0	0	0	0	0	0	0	0	0	0	0	0	0	0	0	0	0
BR_Aracatuba_07	0.836116	0.163884	0	0	0	0	0	0	0	0	0	0	0	0	0	0	0	0	0	0	0	0	0	0	0	0
BR_Aracatuba_14	0.661017	0	0.338983	0	0	0	0	0	0	0	0	0	0	0	0	0	0	0	0	0	0	0	0	0	0	0
BR_Araguaina_06	0.836345	0.163655	0	0	0	0	0	0	0	0	0	0	0	0	0	0	0	0	0	0	0	0	0	0	0	0
BR_Araguatins_12	0.821026	0.0713392	0.107635	0	0	0	0	0	0	0	0	0	0	0	0	0	0	0	0	0	0	0	0	0	0	0
BR_Belem_10	0.697576	0.302424	0	0	0	0	0	0	0	0	0	0	0	0	0	0	0	0	0	0	0	0	0	0	0	0
BR_BVista_10	0.95	0	0.05	0	0	0	0	0	0	0	0	0	0	0	0	0	0	0	0	0	0	0	0	0	0	0
BR_Campinas_04	0.55463	0.201477	0	0.177239	0.066654	0	0	0	0	0	0	0	0	0	0	0	0	0	0	0	0	0	0	0	0	0
BR_Campinas_14	0.813605	0	0.186395	0	0	0	0	0	0	0	0	0	0	0	0	0	0	0	0	0	0	0	0	0	0	0
BR_Campos_03	0.589226	0.225963	0	0.184811	0	0	0	0	0	0	0	0	0	0	0	0	0	0	0	0	0	0	0	0	0	0
BR_CFrio_02	0.636364	0.0689655	0.294671	0	0	0	0	0	0	0	0	0	0	0	0	0	0	0	0	0	0	0	0	0	0	0
BR_CFrio_08	0.169469	0.127803	0.556054	0.146674	0	0	0	0	0	0	0	0	0	0	0	0	0	0	0	0	0	0	0	0	0	0
BR_CGrande_10	0.0575758	0	0.851515	0.0909091	0	0	0	0	0	0	0	0	0	0	0	0	0	0	0	0	0	0	0	0	0	0
BR_DCaxias_01	0.459251	0.365721	0	0.175028	0	0	0	0	0	0	0	0	0	0	0	0	0	0	0	0	0	0	0	0	0	0
BR_DCaxias_10	0.381902	0	0.618098	0	0	0	0	0	0	0	0	0	0	0	0	0	0	0	0	0	0	0	0	0	0	0
BR_FIguacu_06	0.664865	0.335135	0	0	0	0	0	0	0	0	0	0	0	0	0	0	0	0	0	0	0	0	0	0	0	0
BR_Fortaleza_04	0.764045	0.235955	0	0	0	0	0	0	0	0	0	0	0	0	0	0	0	0	0	0	0	0	0	0	0	0
BR_Goiania_10	0.581382	0.154866	0.263752	0	0	0	0	0	0	0	0	0	0	0	0	0	0	0	0	0	0	0	0	0	0	0
BR_GValadares_11	0.53966	0.0641072	0.396233	0	0	0	0	0	0	0	0	0	0	0	0	0	0	0	0	0	0	0	0	0	0	0
BR_Itacoatiara_15	1	0	0	0	0	0	0	0	0	0	0	0	0	0	0	0	0	0	0	0	0	0	0	0	0	0
BR_Itaperuna_02	0.555784	0.444216	0	0	0	0	0	0	0	0	0	0	0	0	0	0	0	0	0	0	0	0	0	0	0	0
BR_Macapa_14	0.860433	0.139567	0	0	0	0	0	0	0	0	0	0	0	0	0	0	0	0	0	0	0	0	0	0	0	0
BR_Maceio_09	0.712553	0.287447	0	0	0	0	0	0	0	0	0	0	0	0	0	0	0	0	0	0	0	0	0	0	0	0
BR_Manaus_09	1	0	0	0	0	0	0	0	0	0	0	0	0	0	0	0	0	0	0	0	0	0	0	0	0	0
BR_Maraba_10	0.830186	0.0970813	0	0.0727329	0	0	0	0	0	0	0	0	0	0	0	0	0	0	0	0	0	0	0	0	0	0
BR_Marilia_04	0.576512	0.24911	0	0.174377	0	0	0	0	0	0	0	0	0	0	0	0	0	0	0	0	0	0	0	0	0	0
BR_Marilia_11	1	0	0	0	0	0	0	0	0	0	0	0	0	0	0	0	0	0	0	0	0	0	0	0	0	0
BR_Marilia_14	0.672193	0.181742	0.146065	0	0	0	0	0	0	0	0	0	0	0	0	0	0	0	0	0	0	0	0	0	0	0
BR_MClaros_06	0.592979	0.407021	0	0	0	0	0	0	0	0	0	0	0	0	0	0	0	0	0	0	0	0	0	0	0	0
BR_Mossoro_09	0.578644	0.357672	0	0	0.0636838	0	0	0	0	0	0	0	0	0	0	0	0	0	0	0	0	0	0	0	0	0
BR_Mossoro_11	0.554133	0.445867	0	0	0	0	0	0	0	0	0	0	0	0	0	0	0	0	0	0	0	0	0	0	0	0
BR_NIguacu_03	0.331391	0.0960526	0	0.410526	0.16203	0	0	0	0	0	0	0	0	0	0	0	0	0	0	0	0	0	0	0	0	0
BR_NIguacu_09	0.355133	0.0631179	0.581749	0	0	0	0	0	0	0	0	0	0	0	0	0	0	0	0	0	0	0	0	0	0	0
BR_Niteroi_01	0.679926	0.257249	0	0	0.0628253	0	0	0	0	0	0	0	0	0	0	0	0	0	0	0	0	0	0	0	0	0
BR_Oiapoque_14	0.211099	0	0.788901	0	0	0	0	0	0	0	0	0	0	0	0	0	0	0	0	0	0	0	0	0	0	0
BR_Pacaraima_11	0.766484	0	0.233516	0	0	0	0	0	0	0	0	0	0	0	0	0	0	0	0	0	0	0	0	0	0	0
BR_Palmas_05	0.708183	0.157187	0	0	0.134629	0	0	0	0	0	0	0	0	0	0	0	0	0	0	0	0	0	0	0	0	0
BR_Palmas_12	0.784863	0	0.215137	0	0	0	0	0	0	0	0	0	0	0	0	0	0	0	0	0	0	0	0	0	0	0
BR_Parnaiba_05	0.2375	0.2625	0	0.5	0	0	0	0	0	0	0	0	0	0	0	0	0	0	0	0	0	0	0	0	0	0
BR_Parnamirim_09	0.472377	0.324777	0	0.202846	0	0	0	0	0	0	0	0	0	0	0	0	0	0	0	0	0	0	0	0	0	0
BR_PPrudente_14	0.675385	0.0618803	0.262735	0	0	0	0	0	0	0	0	0	0	0	0	0	0	0	0	0	0	0	0	0	0	0
BR_RBranco_05	0.202748	0	0	0.144008	0.653245	0	0	0	0	0	0	0	0	0	0	0	0	0	0	0	0	0	0	0	0	0
BR_RBranco_11	1	0	0	0	0	0	0	0	0	0	0	0	0	0	0	0	0	0	0	0	0	0	0	0	0	0
BR_RibPreto_09	0.642887	0.129822	0.227292	0	0	0	0	0	0	0	0	0	0	0	0	0	0	0	0	0	0	0	0	0	0	0
BR_RibPreto_11	0.406125	0	0.367125	0.22675	0	0	0	0	0	0	0	0	0	0	0	0	0	0	0	0	0	0	0	0	0	0
BR_RibPreto_14	0.570165	0	0.429835	0	0	0	0	0	0	0	0	0	0	0	0	0	0	0	0	0	0	0	0	0	0	0
BR_Santarem_10	0	0	0	0	0	0	0	0	0	0	0	0	0	0	0	0	0	0	0	0	0	0	0	0	0	0
BR_SBarbara_08	0	0	0	1	0	0	0	0	0	0	0	0	0	0	0	0	0	0	0	0	0	0	0	0	0	0
BR_SJRPreto_08	0.635204	0.164541	0.0960277	0.104227	0	0	0	0	0	0	0	0	0	0	0	0	0	0	0	0	0	0	0	0	0	0
BR_SJRPreto_14	0.593954	0.100398	0.305648	0	0	0	0	0	0	0	0	0	0	0	0	0	0	0	0	0	0	0	0	0	0	0
BR_Urca_15	0.56597	0	0.264146	0.169884	0	0	0	0	0	0	0	0	0	0	0	0	0	0	0	0	0	0	0	0	0	0
BR_VVelha_06	0.602041	0.193878	0.204082	0	0	0	0	0	0	0	0	0	0	0	0	0	0	0	0	0	0	0	0	0	0	0
CA_Yaounde_14	0	0	0	0	0	0	0	0	0.371966	0	0	0	0	0	0.253331	0	0	0.160705	0	0.13278	0	0	0	0	0.0812192	0
CO_Cali_13	0.475008	0.123705	0.401287	0	0	0	0	0	0	0	0	0	0	0	0	0	0	0	0	0	0	0	0	0	0	0
DO_Dominica_09	0.332162	0.138108	0.52973	0	0	0	0	0	0	0	0	0	0	0	0	0	0	0	0	0	0	0	0	0	0	0
GB_Bijagos_09	0	0	0	0	0	0.4871	0	0	0	0	0	0	0	0.258772	0	0	0	0	0.139577	0	0	0.114551	0	0	0	0
HA_Haiti_10	0.534362	0	0.234845	0.230793	0	0	0	0	0	0	0	0	0	0	0	0	0	0	0	0	0	0	0	0	0	0
KE_Nairobi_12	0	0	0	0.673572	0.23666	0	0	0	0	0	0	0	0	0	0	0	0	0	0	0	0	0	0	0.0897677	0	0
MX_Amacuzac_14	0.343035	0	0.656965	0	0	0	0	0	0	0	0	0	0	0	0	0	0	0	0	0	0	0	0	0	0	0
MX_Iguala_12	0.502102	0	0.497898	0	0	0	0	0	0	0	0	0	0	0	0	0	0	0	0	0	0	0	0	0	0	0
MX_Mazatan_12	1	0	0	0	0	0	0	0	0	0	0	0	0	0	0	0	0	0	0	0	0	0	0	0	0	0
PH_Cbu_13	0	0	0	0.587261	0	0	0.412739	0	0	0	0	0	0	0	0	0	0	0	0	0	0	0	0	0	0	0
SA_Jeddah_12	0.702722	0	0	0	0	0	0.297278	0	0	0	0	0	0	0	0	0	0	0	0	0	0	0	0	0	0	0
SN_Goudiri_12	0	0	0	0.363255	0	0.149247	0	0	0	0	0	0	0.260599	0	0	0	0	0	0	0	0.116788	0	0.11011	0	0	0
SN_Ngari_12	0	0	0	0	0	0	0	0.386872	0	0.0771112	0.536017	0	0	0	0	0	0	0	0	0	0	0	0	0	0	0
SN_Sedhiou_12	0.279602	0	0.144277	0	0	0.181654	0	0	0.0846364	0.124862	0	0	0	0	0	0	0.184968	0	0	0	0	0	0	0	0	0
TH_Bangkok_13	0.908813	0	0	0	0	0	0.0911875	0	0	0	0	0	0	0	0	0	0	0	0	0	0	0	0	0	0	0
UG_Lunyo_13	0	0	0	0	0	0.153846	0	0.140271	0	0	0	0.396682	0	0	0	0.230769	0	0	0	0	0	0	0	0	0	0.0784314
US_California_13	0	0	1	0	0	0	0	0	0	0	0	0	0	0	0	0	0	0	0	0	0	0	0	0	0	0
US_Hawaii_09	0.48719	0	0	0.51281	0	0	0	0	0	0	0	0	0	0	0	0	0	0	0	0	0	0	0	0	0	0
US_NOrleans_12	0.283406	0	0.716594	0	0	0	0	0	0	0	0	0	0	0	0	0	0	0	0	0	0	0	0	0	0	0
US_PuertoRico_14	0.136498	0	0.863502	0	0	0	0	0	0	0	0	0	0	0	0	0	0	0	0	0	0	0	0	0	0	0
US_Tucson_12	0.687525	0	0	0.312475	0	0	0	0	0	0	0	0	0	0	0	0	0	0	0	0	0	0	0	0	0	0
VE_Zulia_04	0.325848	0	0	0.674152	0	0	0	0	0	0	0	0	0	0	0	0	0	0	0	0	0	0	0	0	0	0

Supplementary Table S4

IIIS6 haplotype frequencies

Populations*	3S6_00	3S6_01	3S6_02	3S6_03	3S6_04	3S6_05	3S6_06	3S6_07	3S6_08	3S6_09	3S6_10	3S6_11	3S6_12	3S6_13	3S6_14	3S6_15	3S6_16	3S6_17
AU_Cairns_13	1	0	0	0	0	0	0	0	0	0	0	0	0	0	0	0	0	0
AU_Townsville_09	1	0	0	0	0	0	0	0	0	0	0	0	0	0	0	0	0	0
BR_Aracaju_06	0.711075	0.0811075	0.207818	0	0	0	0	0	0	0	0	0	0	0	0	0	0	0
BR_Aracaju_12	0.479452	0.520548	0	0	0	0	0	0	0	0	0	0	0	0	0	0	0	0
BR_Aracatuba_04	0.83013	0.16987	0	0	0	0	0	0	0	0	0	0	0	0	0	0	0	0
BR_Aracatuba_07	0.627169	0.372831	0	0	0	0	0	0	0	0	0	0	0	0	0	0	0	0
BR_Araguaina_12	0.248493	0.751507	0	0	0	0	0	0	0	0	0	0	0	0	0	0	0	0
BR_Araguatins_12	0.43466	0.56534	0	0	0	0	0	0	0	0	0	0	0	0	0	0	0	0
BR_Campinas_04	0.88466	0	0.11534	0	0	0	0	0	0	0	0	0	0	0	0	0	0	0
BR_Campinas_14	0.344581	0.655419	0	0	0	0	0	0	0	0	0	0	0	0	0	0	0	0
BR_Campos_03	0.720482	0.123695	0.155823	0	0	0	0	0	0	0	0	0	0	0	0	0	0	0
BR_CFrio_02	0.530488	0	0.469512	0	0	0	0	0	0	0	0	0	0	0	0	0	0	0
BR_CFrio_08	0.357537	0.579044	0.0634191	0	0	0	0	0	0	0	0	0	0	0	0	0	0	0
BR_DCaxias_01	0.862079	0	0.137921	0	0	0	0	0	0	0	0	0	0	0	0	0	0	0
BR_DCaxias_10	0.170909	0.76	0	0.0690909	0	0	0	0	0	0	0	0	0	0	0	0	0	0
BR_Goiania_10	0.378205	0.621795	0	0	0	0	0	0	0	0	0	0	0	0	0	0	0	0
BR_GValadares_11	0.24239	0.75761	0	0	0	0	0	0	0	0	0	0	0	0	0	0	0	0
BR_Itacoatiara_15	0	1	0	0	0	0	0	0	0	0	0	0	0	0	0	0	0	0
BR_Itaperuna_02	0.941176	0	0.0588235	0	0	0	0	0	0	0	0	0	0	0	0	0	0	0
BR_Macapa_14	0.374003	0.625997	0	0	0	0	0	0	0	0	0	0	0	0	0	0	0	0
BR_Maceio_09	0.720905	0.279095	0	0	0	0	0	0	0	0	0	0	0	0	0	0	0	0
BR_Manaus_09	0.435065	0.493506	0	0.0714286	0	0	0	0	0	0	0	0	0	0	0	0	0	0
BR_Maraba_10	0.406696	0.593304	0	0	0	0	0	0	0	0	0	0	0	0	0	0	0	0
BR_Marilia_04	0.860049	0	0.139951	0	0	0	0	0	0	0	0	0	0	0	0	0	0	0
BR_Marilia_11	0.0741688	0.925831	0	0	0	0	0	0	0	0	0	0	0	0	0	0	0	0
BR_Marilia_14	0.742491	0.257509	0	0	0	0	0	0	0	0	0	0	0	0	0	0	0	0
BR_MClaros_06	0.945111	0.0548885	0	0	0	0	0	0	0	0	0	0	0	0	0	0	0	0
BR_Mossoro_09	0.860588	0.139412	0	0	0	0	0	0	0	0	0	0	0	0	0	0	0	0
BR_Mossoro_11	0.863636	0.136364	0	0	0	0	0	0	0	0	0	0	0	0	0	0	0	0
BR_NIguacu_03	0.686667	0	0.313333	0	0	0	0	0	0	0	0	0	0	0	0	0	0	0
BR_NIguacu_09	0.26393	0.73607	0	0	0	0	0	0	0	0	0	0	0	0	0	0	0	0
BR_Niteroi_01	0.943966	0.0560345	0	0	0	0	0	0	0	0	0	0	0	0	0	0	0	0
BR_Oiapoque_14	0.133028	0.866972	0	0	0	0	0	0	0	0	0	0	0	0	0	0	0	0
BR_Pacaraima_11	0.100503	0.899497	0	0	0	0	0	0	0	0	0	0	0	0	0	0	0	0
BR_Palmas_05	0.753363	0.246637	0	0	0	0	0	0	0	0	0	0	0	0	0	0	0	0
BR_Palmas_12	0.229906	0.770094	0	0	0	0	0	0	0	0	0	0	0	0	0	0	0	0
BR_Parnaiba_05	0.931957	0	0	0	0	0	0	0	0	0	0	0	0	0	0	0	0	0.0680428
BR_Parnamirim_09	0.782245	0.0971524	0.120603	0	0	0	0	0	0	0	0	0	0	0	0	0	0	0
BR_PPrudente_14	0.216146	0.783854	0	0	0	0	0	0	0	0	0	0	0	0	0	0	0	0
BR_RBranco_05	0.703704	0.222222	0.0740741	0	0	0	0	0	0	0	0	0	0	0	0	0	0	0
BR_RBranco_11	0	1	0	0	0	0	0	0	0	0	0	0	0	0	0	0	0	0
BR_RibPreto_09	0.551661	0.448339	0	0	0	0	0	0	0	0	0	0	0	0	0	0	0	0
BR_RibPreto_11	0.420864	0.517524	0.0616117	0	0	0	0	0	0	0	0	0	0	0	0	0	0	0
BR_RibPreto_14	0	1	0	0	0	0	0	0	0	0	0	0	0	0	0	0	0	0
BR_SBarbara_08	0	0	1	0	0	0	0	0	0	0	0	0	0	0	0	0	0	0
BR_SJRPreto_08	0.680575	0.22624	0.093185	0	0	0	0	0	0	0	0	0	0	0	0	0	0	0
BR_SJRPreto_14	0.417278	0.582722	0	0	0	0	0	0	0	0	0	0	0	0	0	0	0	0
BR_Urca_15	0.245204	0.690048	0.0647482	0	0	0	0	0	0	0	0	0	0	0	0	0	0	0
BR_VVelha_06	0.502984	0.497016	0	0	0	0	0	0	0	0	0	0	0	0	0	0	0	0
CA_Yaounde_14	0	0	0	0	0.224201	0.147865	0	0	0.086514	0	0	0	0.291207	0	0	0.145038	0.105174	0
CO_Cali_13	0	1	0	0	0	0	0	0	0	0	0	0	0	0	0	0	0	0
DO_Dominica_09	0.355408	0.644592	0	0	0	0	0	0	0	0	0	0	0	0	0	0	0	0
GB_Bijagos_09	0.0932331	0	0	0.13985	0.233083	0.279699	0.254135	0	0	0	0	0	0	0	0	0	0	0
HA_Haiti_10	0	0.679214	0	0	0	0	0	0	0	0	0	0.320786	0	0	0	0	0	0
KE_Nairobi_12	0.794717	0	0	0.205283	0	0	0	0	0	0	0	0	0	0	0	0	0	0
MX_Amacuzac_14	0	1	0	0	0	0	0	0	0	0	0	0	0	0	0	0	0	0
MX_Iguala_12	0	1	0	0	0	0	0	0	0	0	0	0	0	0	0	0	0	0
MX_Mazatan_12	0	1	0	0	0	0	0	0	0	0	0	0	0	0	0	0	0	0
PH_Cbu_13	0.511637	0.140419	0.347944	0	0	0	0	0	0	0	0	0	0	0	0	0	0	0
SA_Jeddah_12	0.46947	0.53053	0	0	0	0	0	0	0	0	0	0	0	0	0	0	0	0
SN_Goudiri_12	0.399905	0	0	0.146831	0.158052	0	0.0749672	0.220246	0	0	0	0	0	0	0	0	0	0
SN_Ngari_12	0	0	0	0	0.081796	0.591242	0	0	0	0	0.326962	0	0	0	0	0	0	0
SN_Sedhiou_12	0.241943	0.758057	0	0	0	0	0	0	0	0	0	0	0	0	0	0	0	0
TH_Bangkok_13	0.182957	0.614035	0	0	0	0	0	0	0	0	0	0	0	0.203008	0	0	0	0
UG_Lunyo_13	0	0	0	0	0	0	0.19145	0.188162	0.124589	0.329193	0	0	0	0	0.166606	0	0	0
US_California_13	0	1	0	0	0	0	0	0	0	0	0	0	0	0	0	0	0	0
US_Hawaii_09	0.55015	0	0.44985	0	0	0	0	0	0	0	0	0	0	0	0	0	0	0
US_NOrleans_12	0	1	0	0	0	0	0	0	0	0	0	0	0	0	0	0	0	0
US_PuertoRico_14	0	1	0	0	0	0	0	0	0	0	0	0	0	0	0	0	0	0
US_Tucson_12	1	0	0	0	0	0	0	0	0	0	0	0	0	0	0	0	0	0
VE_Zulia_04	0	0.137147	0.862853	0	0	0	0	0	0	0	0	0	0	0	0	0	0	0

* Name of populations is preceded by the code of the country** and followed by the year of collection (20xx)

** AU (Australia), BR (Brazil), CA (Cameroon), CO (Colombia), DO (Dominica), GB (Gabon), HA (Haiti), KE (Kenya), MX (Mexico), PH (Philippines), AS (Saudi Arabia), SN (Senegal), TH (Thailand), UG (Uganda), US (United States), VE (Venezuela)
