# Supplementary material for: Evolution of kdr haplotypes in worldwide populations of Aedes aegypti: Independent origins of the F1534C kdr mutation
Source: PLoS Negl Trop Dis. 2020 Apr 16;14(4):e0008219. doi: 10.1371/journal.pntd.0008219 (PMC7188295; doi:10.1371/journal.pntd.0008219)
Supplement: S1 Text — (PDF) [file pntd.0008219.s004.pdf]

Supplementary Material S2.

The SeekDeep pipeline consists of three steps: 1) de-multiplex reads and read filtering; 2) clustering of the reads and prediction of the haplotype frequency per each sample; and 3) comparison of haplotypes across all samples. We did the first step using the primers and barcodes described on Supplementary Table S1, keeping only sequences between 360 and 410 nucleotides long after trimming of barcodes and primer sequences (Suppl Table S2).

For quality filtering we kept the sequences with a minimal average Phred quality of 20 within a sliding window of 50 nucleotides moving at 5 nucleotide steps. The software package FastQC (https://www.bioinformatics.babraham.ac.uk/projects/fastqc/) was used to check the sequencing data before and after filtering to ensure we obtained high quality sequencing reads for each sample. After the filtering, only samples that retained at least 100 reads were maintained. We perform the clustering within samples and left out all singlets reads. We also perform interactions until no more collapsing of the formed clusters occurred.

The final step of the pipeline consists of a final result filtering and comparison across all samples. We kept only clusters with at least 10 sequencing reads and used a filter to clean up low frequencies one-off haplotypes at the end of the clustering. The goal was to remove haplotypes with low frequency which may differ from a high frequent haplotype by, for example, only one nucleotide. We also removed all haplotypes that were marked as likely chimeric. Finally, we manually checked each haplotype and performed a Blast search (Altschul et al. 1990) against our reference sequences for those haplotypes with low frequency to ensure that they matched and removed those that did not match.

Altschul SF, Gish W, Miller W, Myers EW, Lipman DJ 1990. Basic local alignment search tool. *J Mol Biol*, 215, 403-410.
