# Supplementary figures and images for: Evolution of kdr haplotypes in worldwide populations of Aedes aegypti: Independent origins of the F1534C kdr mutation
Source: PLoS Negl Trop Dis. 2020 Apr 16;14(4):e0008219. doi: 10.1371/journal.pntd.0008219 (PMC7188295; doi:10.1371/journal.pntd.0008219)

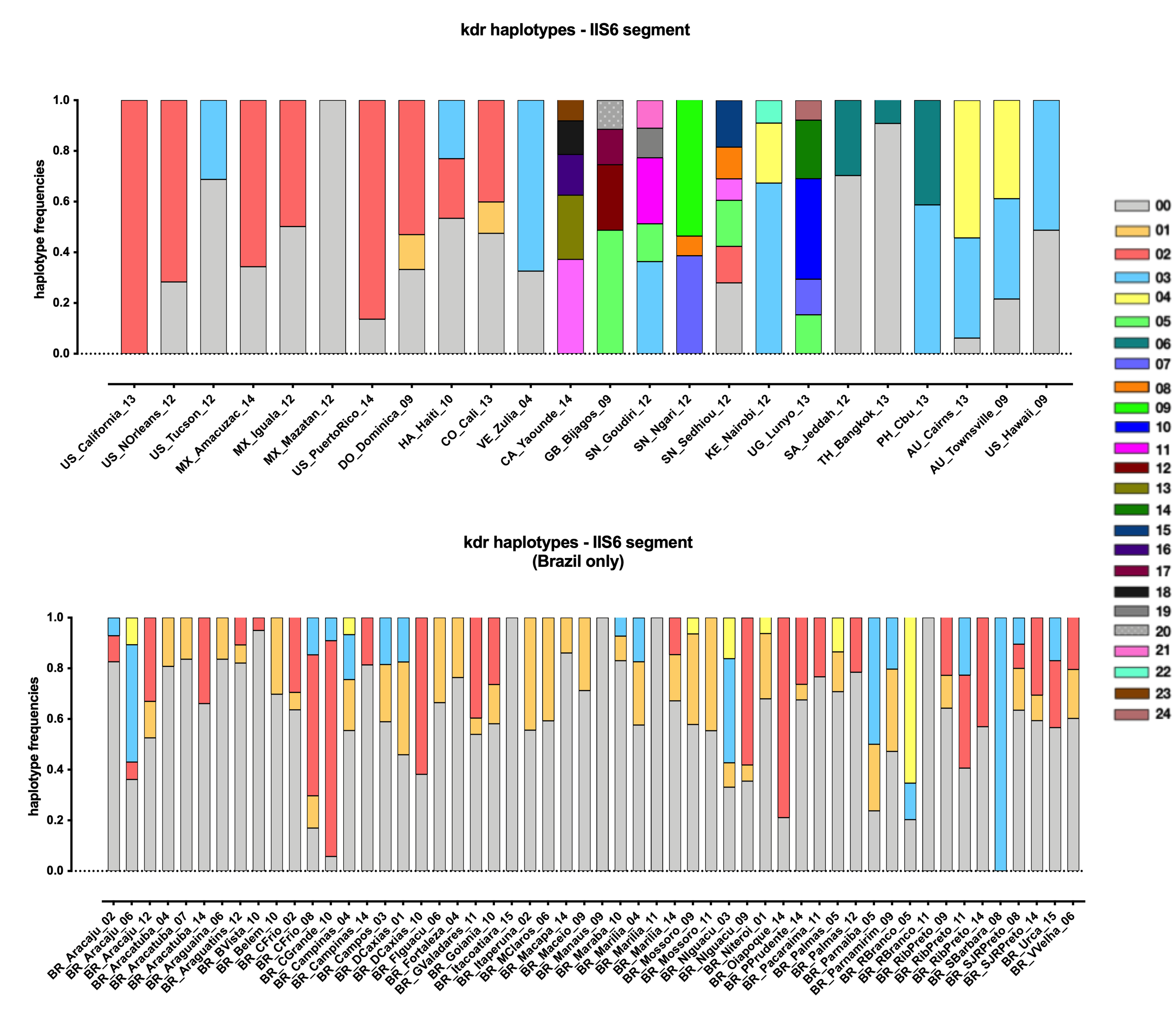

Supplement: S1 Fig — Haplotypic frequencies are displayed for each population (panel above), indicating continent, country and year of collection. The panel bellow shows the populations from Brazil. (TIFF) [file pntd.0008219.s005.tiff]

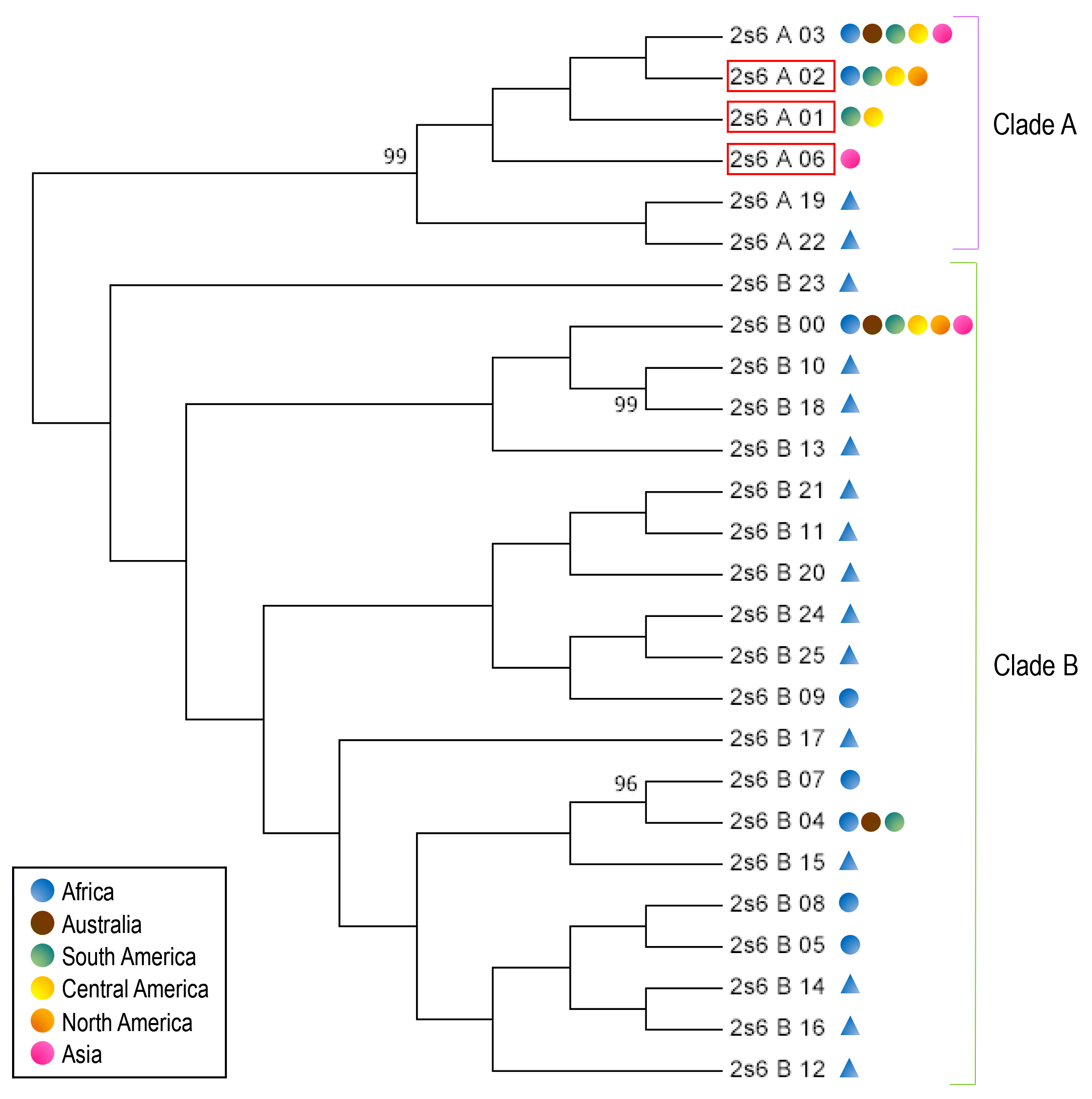

Supplement: S3 Fig — Evolutionary history inferred using Maximum Likelihood with the Tamura-Nei model in MEGA7 [22]. Bootstrap consensus tree (1000 replicates), only. values over 90% are shown. Branch lengths are in scale with the number of substitutions per site. Colored symbols indicate the continent where the haplotypes were found, according to the legend. Haplotypes found in more than one country are in circles and those found exclusively in one country are indicated with a triangular symbol. Haplotypes with non-synonymous substitutions are outlined in red. (TIF) [file pntd.0008219.s007.tif]

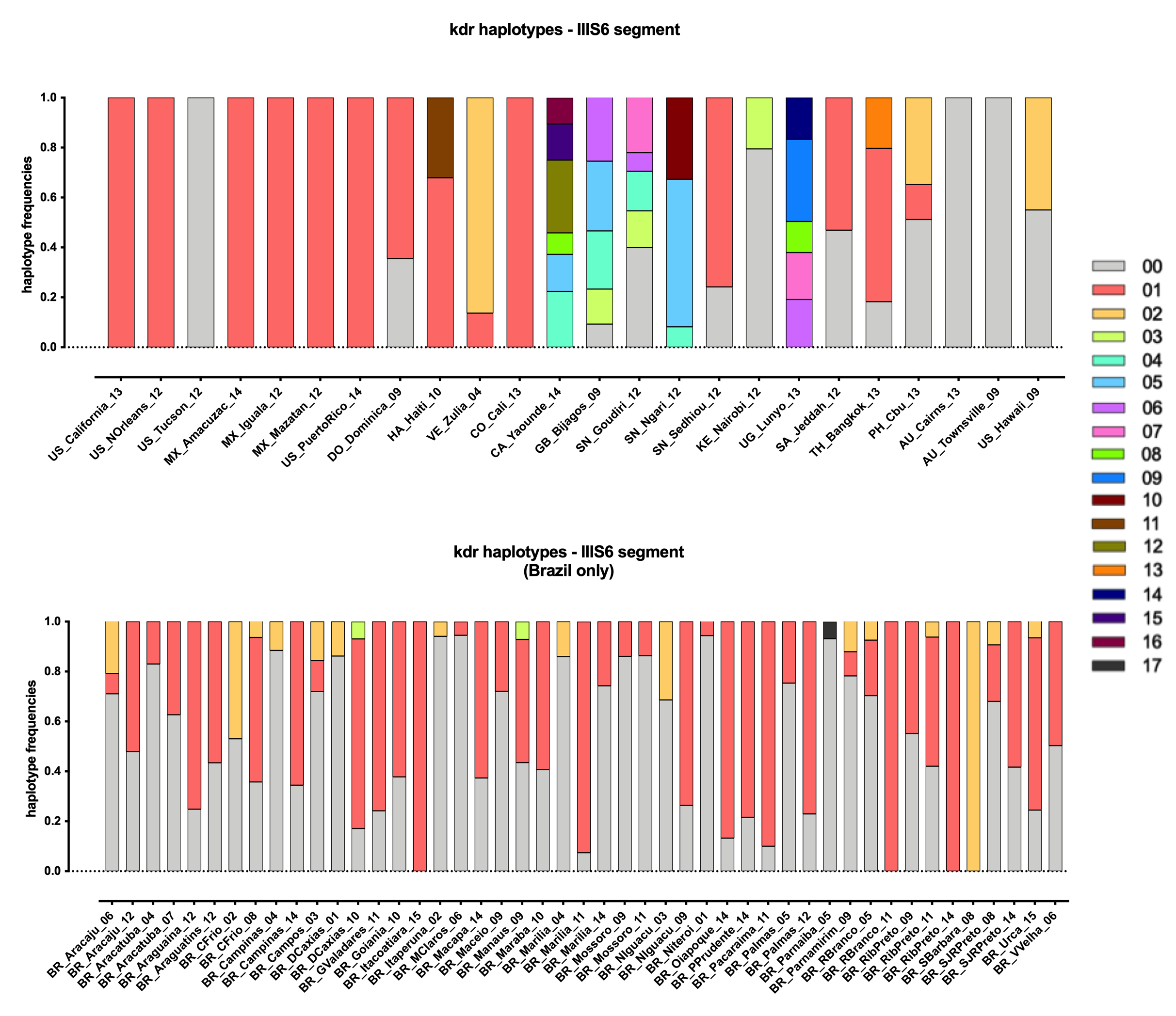

Supplement: S5 Fig — Haplotypic frequencies are displayed for each population (panel above), indicating continent, country and year of collection. The panel bellow shows the populations from Brazil. (TIFF) [file pntd.0008219.s009.tiff]

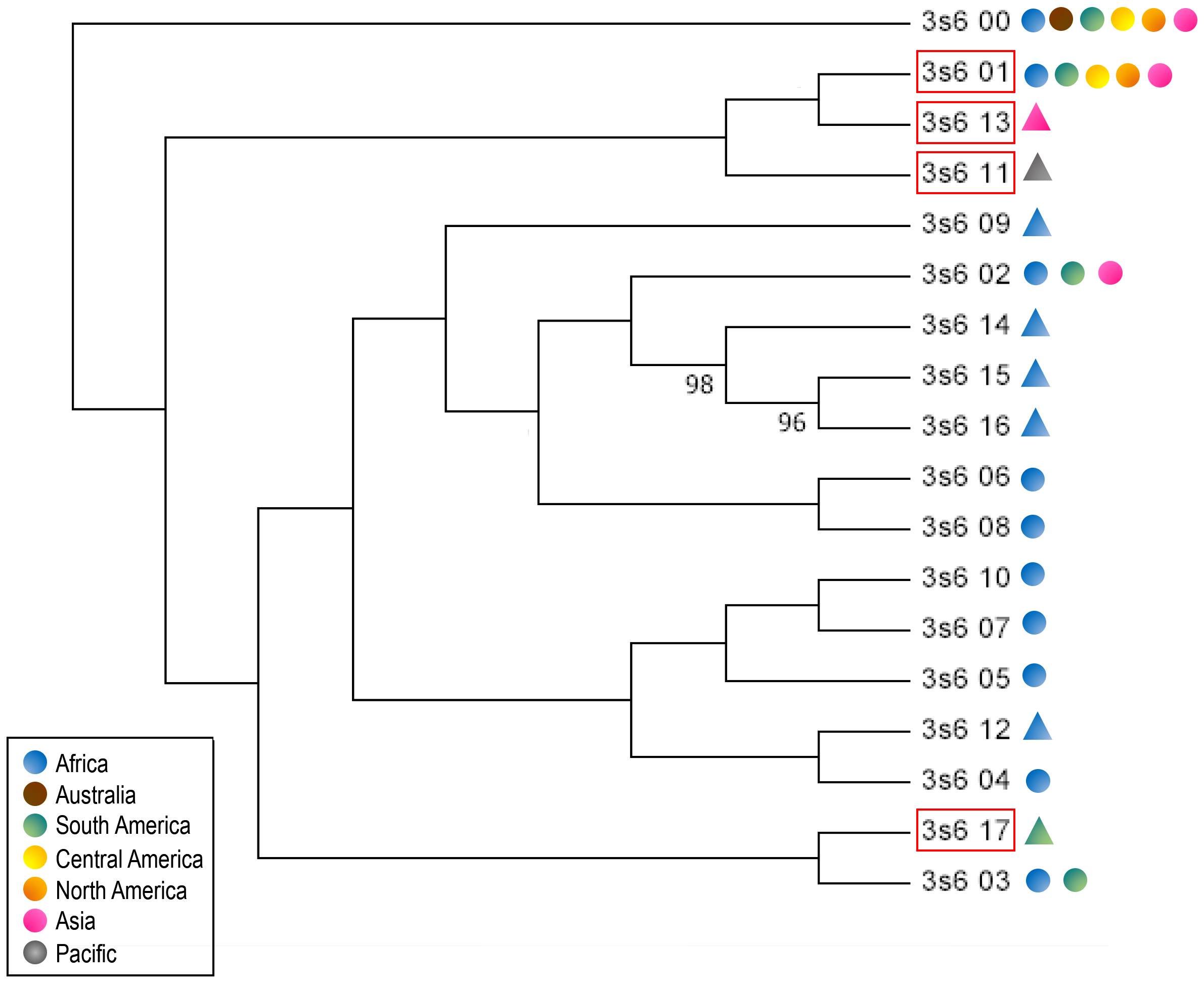

Supplement: S6 Fig — This tree is the bootstrap consensus tree (1000 replicates) inferred by Maximum Likelihood and Tamura-Nei model using MEGA7 software [22], rooted on the 3s6_00 haplotype. Bootstraps values over 90% are shown and branch lengths are in scale with the number of substitutions per site. Colored symbols indicate the continent where the haplotypes were found, according to the legend. Haplotypes found in more than one country are in circles and those found exclusively in one country are indicated with a triangular symbol. Red lined squares denote haplotypes with non-synonymous substitutions. (TIF) [file pntd.0008219.s010.tif]

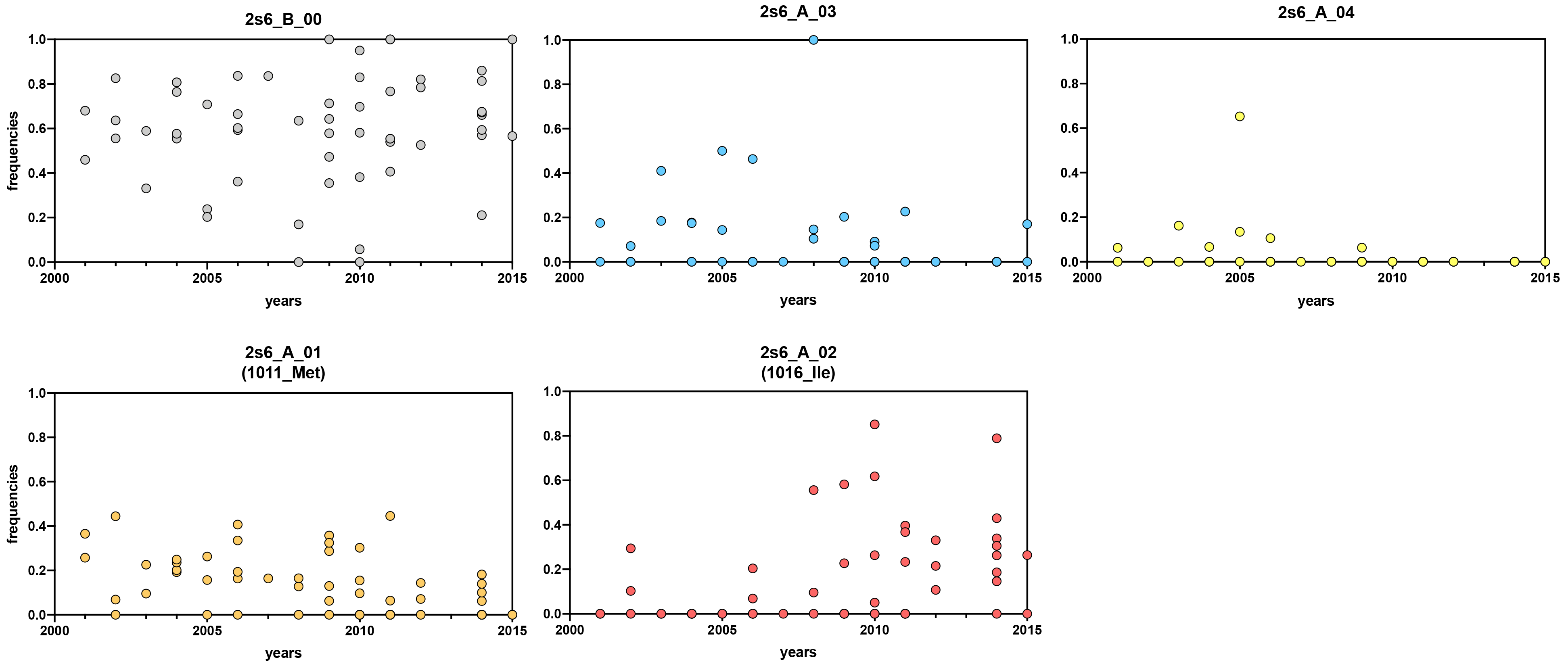

Supplement: S7 Fig — Each dot represents the haplotype frequency for a given population (see S3 Table). The frequencies of the haplotypes from IIS6 segment. (TIF) [file pntd.0008219.s011.tif]

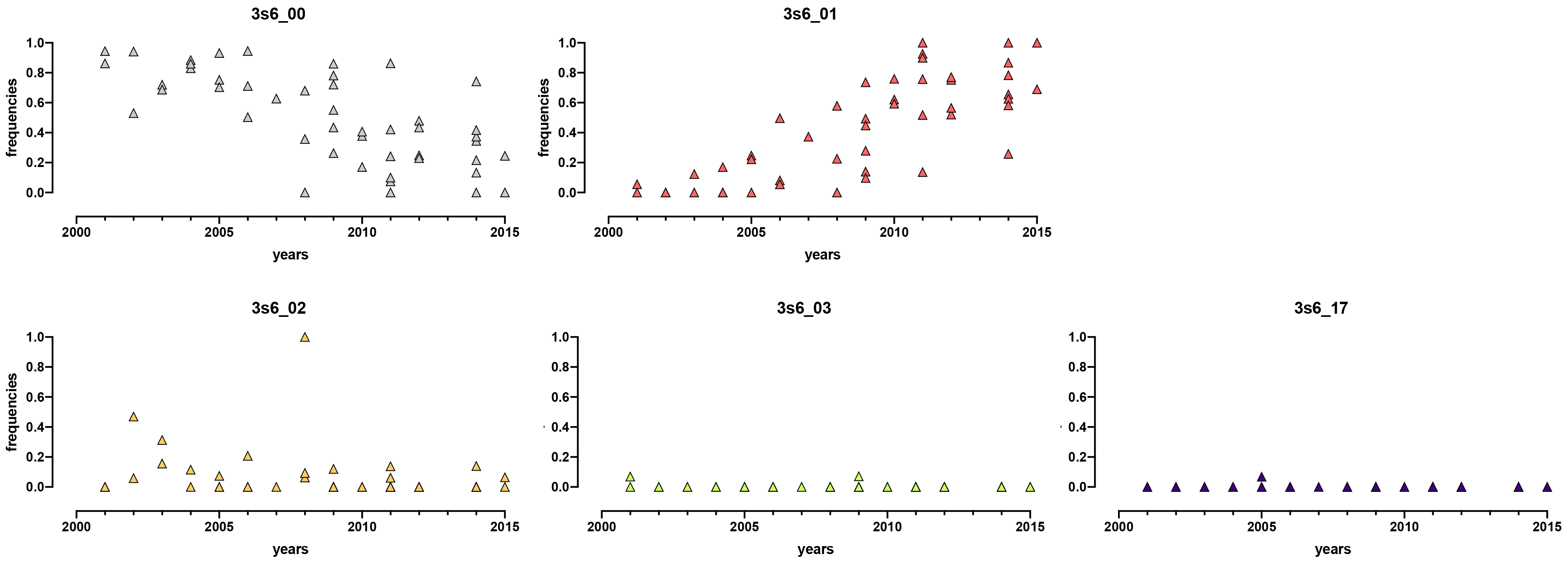

Supplement: S8 Fig — Each dot represents the haplotype frequency for a given population (see S3 Table). The frequencies of the haplotypes from IIIS6 segment. (TIF) [file pntd.0008219.s012.tif]
