# Supplementary material for: Evolution of kdr haplotypes in worldwide populations of Aedes aegypti: Independent origins of the F1534C kdr mutation
Source: PLoS Negl Trop Dis. 2020 Apr 16;14(4):e0008219. doi: 10.1371/journal.pntd.0008219 (PMC7188295; doi:10.1371/journal.pntd.0008219)
Supplement: S2 Fig — Nucleotides in the exon and intron regions are in upper- and lower-case, respectively. Dots indicate the same nucleotides as in the 2S6_B_00 haplotype, whereas nonsynonymous changes are in red. The amino acid translation is indicated above the alignment, with changes numbered according to the NaV protein of Musca domestica, as usual. (PDF) [file pntd.0008219.s006.pdf]

Supplementary Material S6

Alignment of nucleotide sequences of IIS6 haplotypes

```
          <----------------------------------- exon 20 -------------------------------------------|-------------- intron 20 -------->
                  989                                                                 1011
                   |                                                                    |
              I  E S/P M  W  D  C  M  L  V  G  D  V  S  C  I  P  F  F  L  A  T  V  V I/M G  N  L  V
#2s6_A_03 GATCGAATCCATGTGGGATTGTATGCTTGTGGGTGACGTGTCCTGTATTCCGTTCTTTTTGGCCACCGTAGTGATAGGAAATCTAGTagtaagtattccgtttgggagttcttctataag [120]
#2s6_A_01 ...........................................................................G............................................
#2s6_A_02 ........................................................................................................................
#2s6_A_06 .......C................................................................................................................
#2s6_A_19 ..........................................T.............................................................................
#2s6_A_22 ........................................................................................................................
#2s6_B_00 .........................................................................................................a.....a...g....
#2s6_B_04 ...............................................................................................................a........
#2s6_B_05 ........................................................................................................................
#2s6_B_07 ...............................................................................................................a........
#2s6_B_08 ........................................................................................................................
#2s6_B_09 ........................................................................................................................
#2s6_B_10 ....................................................................................................c...................
#2s6_B_11 ........................................................................................................................
#2s6_B_12 ...............................................................................................................a........
#2s6_B_13 ...............................................................................................................a........
#2s6_B_14 ........................................................................................................................
#2s6_B_15 ...............................................................................................................a........
#2s6_B_16 ........................................................................................................................
#2s6_B_17 ........................................................................................................................
#2s6_B_18 ...............................................................................................................a........
#2s6_B_20 .........................................................................................................a.....a...g....
#2s6_B_21 .....................C..................................................................................................
#2s6_B_23 ...............................................................................................................a........
#2s6_B_24 ................................................A.....................................................c........a...g....
#2s6_B_25 ...........................................................................................t............................

          <------------------------------------------------ intron 20 ------------------------------------------------------------->

#2s6_A_03 gctgactgaaagtaaattggagcgcacaacaagacctgttatgctgtaagttccagcactaaatttctcaggttgaattgcagtagttcaatcgaaatctcgaactttcattttgataac [240]
#2s6_A_01 ........................................................................................................................
#2s6_A_02 ........................................................................................................................
#2s6_A_06 ........................................................................................................................
#2s6_A_19 .......................................................................................................g...............a
#2s6_A_22 .......................................................................................................g................
#2s6_B_00 ..............................-......a...........t.cgt---------------------G...ca.c.....ac.---...----..c.g..g..c......g.
#2s6_B_04 ........g.............t.......-....g.a...........t.cgt---------------------G...ca.c.....a.----..T----..c.g..g..c......g.
#2s6_B_05 ..............................-......a...........t.cgt---------------------G...ca.c.....a..---...----..c.g..g..c......g.
#2s6_B_07 ........g.............t.......-......a...........t.cgt---------------------G...ca.c.....a..---...----..c.g..g..c......g.
#2s6_B_08 ........................t.....-......a...........t.cgt---------------------G...ca.c.....a..---...----..c.g..g..c......g.
#2s6_B_09 ..............................-......a...........t.cgt---------------------G...ca.c.....a..---...----..c.GA.g..c......g.
#2s6_B_10 ..............................-.cca..a...........t.cgt---------------------G...ca.c.....a..---...----.GC.g..g..c......g.
#2s6_B_11 ..............................-......a...........t.cgt---------------------G...ca.c.....a..---...----..c.g..g..c......g.
#2s6_B_12 ..............................-......a...........t.cgt---------------------G...ca.c.....a..---...----..c.g..g..c......g.
#2s6_B_13 ..............................-g.....a...........t.cgt---------------------G...CA.-------------------..c.g..g..c......g.
#2s6_B_14 ..............................-......a...........t.cgt---------------------G...ca.c.....a..---...----..c.g..g..c......g.
#2s6_B_15 ........g.............t..........................t.cgt---------------------G...CA.a.....a..---...----..c.g..g..c......g.
#2s6_B_16 ..............................-......a...........t.cgt---------------------G...CA.a.....a..---...----..c.g..g..c......g.
#2s6_B_17 ..............................-...t..a...........t.cgt---------------------G...ca.c.....a..---...----T.c.a..g..c......g.
#2s6_B_18 ..............................-.cca..a...........t.cgt---------------------G...ca.c.....a..---...----.GC.g..g..c......g.
#2s6_B_20 ..............................-......a...........t.cgt---------------------G...ca.c.....a..---...----..c.g..g..c......g.
#2s6_B_21 ..............................-......a...........t.cgt---------------------G...ca.c.....a..---...----T.c.a..g..c......g.
#2s6_B_23 ..............................-......a...........t.cgt---------------------G...ca.c.....a..---...----T.c.g..g..c....c.g.
#2s6_B_24 .....g........................-......a...........t.cgt---------------------G...ca.t...a.a..---...----..-.g..g..c......g.
#2s6_B_25 ..............................-......a...........t.cgt---------------------G...ca.c.....a..---...----..c.g..g..c......g.
```

```
         <-------------------------------- intron 20 -------------------------------------------------------------------|-- exon 21--->
                                                                                                                         1016
                                                                                                                          |
                                                                                                                          V  L  N  L
#2s6_A_03 agcaatactaga-------------cgcgcatagaacatacaaatttacatatagtcagcctttcatgcattctatcgtgctaaccgacaaattgtttcccacccgcacagGTACTTAACCTTTT  [365]
#2s6_A_01 ............-------------....................................................................................................
#2s6_A_02 ............-------------......................................................................................A.............
#2s6_A_06 ............-------------....................................................................................................
#2s6_A_19 ............-------------........................................t...............................................G...........
#2s6_A_22 ............-------------........................................t...........................................................
#2s6_B_00 .t.....t....ggcgtgctagcag..a..gag.gg.g...c.......t.t.......t.....t........t............................t.....................
#2s6_B_04 .t...c......ggcgtgctagcag..a..gag.gg.g...c.......t.t.g...........t...........................................................
#2s6_B_05 .t...c......ggcgtgctagcag..a..gag.gg.g...c.......t.t.............t.......g...a...........t...................................
#2s6_B_07 .t...c......ggcgtgctagcag..a..gag.gg.g...c.......t.t.g...........t...........................................................
#2s6_B_08 .t...c......ggcgtgctagcag..a..gag.gg.g...c.......t.t.............t.......g...a...........t...................................
#2s6_B_09 .t...c.....tggcgtgctagcag..a..gag.gg.g...c....a..t.t.............t......................a....................................
#2s6_B_10 .t.....t....ggcgtgctagcag..a..gag.gg.g...c.......t.t.......t.....t.......g...................................................
#2s6_B_11 .t...c....a.ggcgtgctagcag..a..gag.gg.g...c.......t.t.............t...........................................................
#2s6_B_12 .t...c......ggcgtgctagcag..a..gag.gg.g...c.......t.t.............t.......g...a...........t...................................
#2s6_B_13 .t...c......ggcgtgctagcag..a..gag.gg.g...c.......t.t.............t...................t.................t.....................
#2s6_B_14 .t...c......ggcgtgctagcag..a..gag.gg.g...c.......t.t............at.......g...a...........t...................................
#2s6_B_15 .t...c......ggcgtgctagcag..a..gag.gg.g...c.......t.t.............t.......g...a...........t...................................
#2s6_B_16 .t...c......ggcgtgctagcag..a..gag.gg.g...c.......t.t..a..........t.......g...a...........t...................................
#2s6_B_17 .t...c......ggcgtgctagcag..a..gag.gg.g...c.......t.ta............t...........a...............................................
#2s6_B_18 .t.....t....ggcgtgctagcag..a..gag.gg.g...c.......t.t.......t.....t.......g...................................................
#2s6_B_20 .t...c....a.ggcgtgctagcag..a..gag.gg.g...c.......t.t.............t...........................................................
#2s6_B_21 .t...c....a.ggcgtgctagcag..a..gag.gg.g...c.......t.t.............t.a.........................................................
#2s6_B_23 .t...c......ggcgtgctagcag..a..gag.gg.g...c.......t.t.............t...........................................................
#2s6_B_24 .t...c......ggcgtgctagcag..a..gag.gg.g...c.......t.t.............c......a....................................................
#2s6_B_25 .t...c......ggcgtgctagcag..a..gag.gg.g...c.......t.t.............c................................tg.........................
```
