# Supplementary material for: Evolution of kdr haplotypes in worldwide populations of Aedes aegypti: Independent origins of the F1534C kdr mutation
Source: PLoS Negl Trop Dis. 2020 Apr 16;14(4):e0008219. doi: 10.1371/journal.pntd.0008219 (PMC7188295; doi:10.1371/journal.pntd.0008219)
Supplement: S4 Fig — Nucleotides in the exon and intron regions are in upper- and lowercase letters, respectively. Dots indicate the same nucleotides as in the 3S6_00 haplotype. Non-synonymous SNPs changes are in red. The amino acid translation is indicated above the alignment, numbered according to the NaV protein of Musca domestica, as reference. (PDF) [file pntd.0008219.s008.pdf]

Supplementary Material S8
Alignment of nucleotide sequences of IIIS6 haplotypes

```
        <--------------------------------------------------- exon 31 ------------------------------------------------------------>
          1520                                 1532  1534
            |                                   |     |
         E T/I N  I  Y  M  Y  L  Y  F  V  F  F I/T I F/C G  S  F  F  T  L  N  L  F  I  G  V  I  I  D  N  F  N  E  Q  K  K  K  A
#3s6_00 GAGACCAACATCTACATGTACCTCTACTTTGTGTTCTTCATCATCTTCGGGTCGTTCTTCACGCTGAATCTGTTCATCGGTGTCATCATCGACAACTTCAACGAGCAGAAGAAGAAAGCC [120]
#3s6_10 ..........................T..C..........................................................................A...............
#3s6_13 ....T.........................................G.........................................................................
#3s6_01 ..............................................G.........................................................................
#3s6_11 ........................................C...............................................................................
#3s6_09 .............................C..........................................................................................
#3s6_17 .............................C..........................................................................................
#3s6_03 .............................C..........................................................................................
#3s6_12 .............................C..........................................................................................
#3s6_04 .............................C..........................................................................................
#3s6_05 .............................C..........................................................................A...............
#3s6_07 .............................C..........................................................................A...............
#3s6_02 .............................C..........................................................................A...............
#3s6_06 .............................C..........................................................................................
#3s6_08 ........................................................................................................................
#3s6_14 .............................C..........................................................................................
#3s6_15 .............................C..........................................................................................
#3s6_16 .............................C..........................................................................................


        <------------------------------------------ exon 31 ----------------------------------------------------|--intron 31--->
         G  G  S  L  E  M  F  M  T  E  D  Q  K  K  Y  Y  N  A  M  K  K  M  G  S  K  K  P  L  K  A  I  P  R  P  R
#3s6_00 GGTGGCTCACTGGAAATGTTCATGACGGAGGATCAGAAAAAGTACTACAACGCCATGAAAAAGATGGGCTCGAAGAAGCCGCTGAAAGCTATTCCACGGCCTAGGgtaaggcatttccat [240]
#3s6_10 ......................................G.........................................................A.......................
#3s6_13 ........................................................................................................................
#3s6_01 ........................................................................................................................
#3s6_11 ........................................................................................................................
#3s6_09 ........................................................................................................................
#3s6_17 ........................................................................................................................
#3s6_03 ........................................................................................................................
#3s6_12 ......................................G.............................................................................g...
#3s6_04 ......................................G.................................................................................
#3s6_05 ......................................G.................................................................................
#3s6_07 ......................................G.........................................................A.......................
#3s6_02 ......................................G.................................................................................
#3s6_06 ........................................................................................................................
#3s6_08 ........................................................................................................................
#3s6_14 ......................................G.......................................................................a..a....g.
#3s6_15 ..............................................................A...............................................aa.a......
#3s6_16 ..............................................................A.........................................A.....aa.a......
```

```
<----------------------------intron 31---------------------|------------------------exon 32-------------------------------->
                                                                                             1605
                                                                                              |
                                                             W  R  P  Q  A  I  V  F  E  I  V T/A N  K  K  F  D  M  I  I
#3s6_00 cgcacatcaactgtgacgtattccttcctaatctcgctattctcaatttcagTGGCGACCACAAGCAATAGTATTCGAAATAGTTACCAATAAGAAGTTCGACATGATCATCAT [354]
#3s6_10 ..................................................................................................................
#3s6_13 ..................................................................................................................
#3s6_01 ..................................................................................................................
#3s6_11 ..................................................................................................................
#3s6_09 .............................g....................................................................................
#3s6_17 .....................................................................................G............................
#3s6_03 ..................................................................................................................
#3s6_12 ..................................................................................................................
#3s6_04 ..................................................................................................................
#3s6_05 ..................................................................................................................
#3s6_07 ..................................................................................................................
#3s6_02 ..........................t..g.....a.c......c...c.................................................................
#3s6_06 ..........................t..g.....a.c......c...c.................................................................
#3s6_08 ..........................t..g.....a.c......c...c.................................................................
#3s6_14 t-.t.g.......a....a.a.....t........a.c......c...c.................................................................
#3s6_15 t-.t.g............a.......t........a.c......c...c.................................................................
#3s6_16 t-.t.g............a.......t........a.c......c...c.................................................................
```
