# Supplementary material for: Evolution of kdr haplotypes in worldwide populations of Aedes aegypti: Independent origins of the F1534C kdr mutation
Source: PLoS Negl Trop Dis. 2020 Apr 16;14(4):e0008219. doi: 10.1371/journal.pntd.0008219 (PMC7188295; doi:10.1371/journal.pntd.0008219)
Supplement: Attachment — Submitted filename: PNTD-D-19-01832_Response to reviewers.docx [file pntd.0008219.s013.docx]

Rio de Janeiro, February 12^th^ 2020

**(PNTD-D-19-01832)**

**Responses to Editor and reviewers**

Dear Editor of PLOS NTD,

On behalf of all authors, I thank you for the opportunity to submit a revised version of our manuscript entitled “**Evolution of kdr haplotypes in worldwide populations of *Aedes aegypti*: independent origins of the F1534C kdr mutation**”. We thank you for the comments and suggestions raised by the three reviewers, which are addressed point-by-point below. After this review, we believe that our manuscript has significantly improved and is hopefully now suitable for publication in your journal.

1)

Reviewer #1: The study is hypothesis-driven and the methods are appropriate for testing the hypothesis

Reviewer #2: Methodology including NGS, phylogenical tools, targeted markers and sample size is adequate and well described in the paper (including in supp information)

Reviewer #3: The temporal variation paragraph needs to be clarified. When you mention that sequences from Brazilian populations spanning from 2001 to 2015 were evaluated in terms of frequency variation, it is not clear which frequencies are you referring to. Do you mean assessing if frequencies of different NaV haplotypes increase or decrease over time? If that so, please state it more explicitly.

>> Yes, we were referring to the frequencies of each haplotype in samples of different years. The sentence in the Methods section was modified to:

Lines 259-261: “As sequences of several *Ae. aegypti* populations from Brazil spanning 2001 to 2015 were available, we evaluated whether the frequencies of the observed *Na_V_* haplotypes increased or decreased over time in this country.”

2)

Reviewer #1: The results are well-presented

Reviewer #2: yes the analysis match the analysis plan. Results are clearly presented and well illustrated by graphs to facilite reader understanding.  Resolution (quality) of pictures may need to be double check.

>> We are resubmitting all the figures with resolution as required by the journal. However, generally when automatically inserted in the pdf file for revision their quality is low. We suggest that the reviewers could download the figures files.

Reviewer #3: In the Haplotype analyses section it is not clear what you mean by "Nine haplotypes were specific to a single population, six in Africa." Could you please clarify? Does this mean that the 9 haplotypes were all found within population "6" in Africa? Please clarify the language to make this explicit.

>> We thank the reviewer for this observation . What we meant was that 9 haplotypes did not appeared in more than one population, sometimes called “private” alleles. Out of these 9, 6 belonged to an African country. We corrected the sentence in the results section to:

Lines 362-364: “Nine of the haplotypes only occurred in a single population. Out of these nine, six belonged to African populations (Supplementary Table S4)”.

3)

Reviewer #1: The conclusion is supported by their data.  
The limitations of analysis are not discussed `
>> In this specific comment it was hard to exactly address the reviewer comment, since it is unclear to which limitation she/he was referring to. Nevertheless, in our view the biggest challenge is the difficulty of phasing the haplotypes from the two distinct segments in a given population, because when both segments were polymorphic one could not unambiguously identify the haplotypes that phase with each other. This mean that we could phase haplotypes only when one of the two segments was monomorphic in a given population. Although this is a limitation, we do not consider it impacts the interpretation of the results because we have at least six populations that satisfied this condition. We clarified the text in both the methods and results sections that now read as follow:

- Methods (lines 250-255): “Although IIS6 and IIIS6 segments are ~ 44.5 Kb apart, since linkage disequilibrium in *Ae. aegypti* is about 50-80 Kb [distance at which correlation is halved from maximum [23, 24]], we expect variation in the two segments to be correlated. As we could not phase the haplotypes when both segments were polymorphic, haplotypes phase could be determined only in populations monomorphic for one of the two segments.”

- Results (lines 399-403): “We phased IIS6 and IIIS6 to combine the variability from both segments, and thus increase our analysis power. The phasing could only be determined in populations which haplotypes were monomorphic in at least one of the segments, because it is not possible to indicate which haplotypes are on the same chromosome when populations were polymorphic in both segments.”

Reviewer #2: The conclusion supports the data. The discussion section however is a bit too "technical " for me and could be improved to ease reader understanding (mentioning all haplotype names can be boring and difficult to follow). Puting their findings in perspectives of vector control activities in Brazil would be a plus!
>> We agree with the reviewer that the text was too detailed and difficult to follow. We made an effort to name the haplotypes following certain logic (such as 2s or 3s as a prefix, in the case of 2S if belonging to A or B clade, followed by a number; and when haplotypes were phased we tried to indicate the connection between the haplotypes of both segments also in the most logical way we found). Furthermore, the figures were designed to clarify this nomenclature.

In order to improve the reading flow of the discussion, we have divided it into sub-sections one describing the results of the spatial (lines 518-587) and the other the temporal (lines 589-627) analysis. Some additional alterations were made in the discussion section in response to specific comments, as indicated along this response letter.

We have also added a sentence discussing the possible contributions of this work to vector control activities:

- Lines 600-604: “Results observed in our study corroborate the increase of *kdr* frequency in *Ae. aegypti* from Brazil, which in part justify the spread of pyrethroid resistance and support the genotyping tools currently used for surveillance of 1016I and 1534C *kdr* mutations in natural populations [8].”

Reviewer #3: As part of the conclusions it is stated that future work will aim at further elucidating how insecticide resistance is interfering with genetic structure, migration as well as vector competence in local populations of Ae. aegypti. I would suggest to express this differently because it can be argued that population genetic structure is what actually modulates patterns if insecticide resistance, and vector competence. That is, genetically distinct populations may differ in their potential to develop insecticide resistance as well as in vector competence. Most likely insecticide use and population genetics are likely integrating and affecting vectors' evolution.

>> We agree. The sentence was improved:

- Lines 619-622: “Future work will aim at further elucidating specific questions such as how insecticide resistance is evolving in complex landscape scenarios, considering genetic structure, migration as well as vector competence in local populations of *Ae. aegypti*”

4)

Reviewer #1: (No Response)

Reviewer #2: (No Response)

Reviewer #3: In the Abstract/Methods section replace "populations from 15 countries, including South and North America, Africa, Asia, Pacific, and Australia." with "populations from 15 countries in South and North America, Africa, Asia, Pacific, and Australia.

>> Done.

In the Abstract/Conclusion section replace "These results support that some kdr mutations arise de novo and are regionally distributed in Ae. aegypti. However, our results also suggest for the first time that the 1534C kdr mutation had at least two independent origins." with "These results provide evidence of kdr mutations arising de novo at specific location within the Ae. aegypti geographic distribution. In addition, our results suggest that the 1534C kdr mutation had at least two independent origins. "

>> Done.

In the Author Summary Section: Delete "the" before "residences" in the following sentence: "...the class of insecticide most employed worldwide inside and around the residences"

>> Done.


In the Author Summary Section: Change "from a DNA collection" to ""from DNA collections" in this sentence: "By evaluating the sequences of two fragments of the NaV gene, obtained from a DNA collection of Ae. aegypti from several countries,..."

>> Done. Very well noticed. Thank you!


In the Author Summary Section: Tell me first that two independent origins were evidenced for the F1534C kdr mutation, found in American, African and Asian populations and then mention the lack of geographic variation in some Kdr mutations (i.e., positive results first).

>> Agreed. Done.

In the Author Summary Section: Rewrite this sentence: "These results improve the knowledge about insecticide resistance evolution in the mosquito who is presently one of the main actors of global health concerns." to something more like this "Our results clarify insecticide resistance evolution in one of the main vectors of several global diseases"
>> We changed as recommended, although we preferred not to stating that this mosquito is vector of diseases, but of arboviruses.

-Lines 72-73: “Our results increase our knowledge of insecticide resistance evolution in one of the main arboviral mosquito vectors of major global diseases.”

In the Introduction add "worldwide" to this sentence: "Aedes aegypti is considered one of the most successful invasive species, worldwide.."
>> Added.

In Discussion section: Add scientific name for the codling moth 
>> *Cydia pomonella* added.

In Discussion section: When you say "They found the kdr 1534C mutation in individuals with scaling patterns usually found in both subspecies (Aaa and AAf)." It is no clear what "they" is referring to. Change to passive voice or mention the subject to which "they" is referring to for clarity. Apply this to the whole paragraph.

>> Agreed. Modifications were done accordingly:

- Lines 509-520: “The *kdr* 1534C mutation was observed in individuals with scaling patterns usually found in both subspecies (Aaa and AAf). As in our analyses and previous work [38], the IIS6 haplotypes belonged to two distinct clades for IIS6 in those African populations. The *kdr* 1534C African sequences were associated ...”

5)

Reviewer #1: Mutations in two transmembrane regions , IIS6 and IIIS6, of the sodium channel protein have previously been shown to be associated with resistance of Aedes aegypti to pyrethroid insecticides. This manuscript reports a haplotype analysis of the genomic regions encoding IIS6 and IIIS6 in Ae. aegypti populations from 15 countries to understand the evolution of these mutations, known as kdr mutations. This study involved a large amount of sequence data as well as haplotype network and phylogenetic analyses.  They found 26 and 18 haplotypes for IIS6 and IIIS6, respectively, identified previously identified kdr mutations in some of the haplotypes and confirmed specific geographical distributions of these mutations.  Evidence is provided suggesting that one of the mutations, F1534C in IIIS6, arose twice with two different origins.   However, origins of the kdr mutations in IIS6 remain unclear.

Data presented seem solid. I have only the following minor comments:
The authors need to point out that I1532T and 1605A have not been shown to be associated with pyrethroid resistance in Ae. aegypti; However, the role of T1520I in pyrethroid resistance has been functionally confirmed in an in vitro expression system (Chen et al., 2019, PloS NTD)

>> We thank the reviewer for this advice and the reference has been included in the text:

- Lines 556-560: “Electrophysiological assays with distinct *kdr* mutations introduced in an *AaNaV1* cDNA, then expressed in a *Xenopus* oocytes heterologous system, evidenced that the 1520I alone did not alter the channel sensitivity to pyrethroids and DDT. However, resistance to permethrin was higher with 1520I + 1534C than with 1534C alone [44]”

Page 7, 3rd paragraph: 1523T in “The Asian 1523T +1534C haplotype (3s6_13)” should be “1520I.”

>> Yes! Corrected.  

The resolution of Fig. 4 is poor.

>> This probably happened when the figures were merged into the pdf file. As this figure 4 is too large, please, download the figure files separately and check if the resolution is good enough.   


Reviewer #2: Dear Editor,

The authors investigated the origin and dispersion of kdr haplotypes in Ae. aegypti from multiple locations (South and North America, Africa, Asia, Pacific, and Australia) using a combination of NGS and phylogeny approaches. The authors showed that all mutations seemed to be “regionally” distributed while the 1534C kdr mutation seems to be widely distributed worldwide. Their results suggest that most of kdr mutations had single origin (ie “de novo” mutation) and then spread regionally while the 1534C kdr mutation had at least two independent emergence. Although the paper is not  always “easy” to follow (especially the discussion part that contains a lot of haplotype names), this study provides new insight into the selection and spread of insecticide resistant markers in Aedes mosquito and has practical implications for designing more efficient Insecticide Resistance containment plan. The paper is suitable for publication in Plos NTD after minor revision.

Minor revisions;
The discussion is too “technical” because it contains many (unecessary?) abbreviations and haplotype names! The authors should make efforts to summarize the information’s instead of repeating the results. I would advise them also to discuss the results in perspective of vector control activities (eg history of insecticide use in Brazil and occurrence and spread of kdr mutations, how to use their data to better implement IRM ?, etc)

>> We understand the reviewer’s point of view. However, precisely citing the haplotypes was important to distinguish between a single or multiple origins for the *kdr* mutations. As mentioned above (block 3), we struggled to find a manner to describe the haplotypes and their distribution, and named them in what we think is the most logical way to make the evolutionary path we propose easy to follow. We expanded the explanation in the Methods to clearly explain the logic of the adopted naming scheme so that the reader could more easily follow the origin of the haplotypes when reading about then, without needing to go back and forth in the text to understand to which haplotype we are referring to:

- Lines 246-250: “The haplotypes were named as 2s6 or 3s6 (in reference to IIS6 and IIIS6 segments, respectively), followed by an identifying number. As evident in the Results section, the IIS6 haplotypes were grouped into A or B clades. Accordingly, we added A or B, following 2s6, to the names of IIS6 haplotypes.”

- It is true that most of our samples were from Brazil. As these were from collections of distinct years, we took advantage of that and performed a temporal analysis. However, we were evaluating the molecular diversity and distribution of *kdr* haplotypes in a worldwide perspective. In our discussion we tried to review more broadly evidence of the role of *kdr* mutations in resistance. We believe that a discussion focused on *kdr* history in Brazil and implications for the IRM system would be more appropriate for a study exclusively focused on Brazil.

- Several other modifications were performed in the discussion section, as specified along this response letter.

P10, Third paragraph. Interestingly the I1011M mutation seems to decrease over time in Brazil. Since pyrethroid resistance is increasing in Aedes aegypti, do the authors suggest that the I1011M has no link with PYR resistance? If the answer is yes, the authors should be more explicit on this matter.

>> Yes. In all Brazilian samples we previously evaluated, I1011M was related to duplication, and all individuals were heterozygous. We selected a line I1011M by doing specific crosses and maintained that for several generations, such selected line was susceptible to pyrethroid. We included this information:

- Lines 581-582: “An *Ae. aegypti* line selected to carry this mutation was not resistant to deltamethrin [48] ”

P20, fourth paragraph. The absence of double homozygotes for kdr mutations at position 1016 and 1534 has been previously observed in South East Asia especially in Thailand, Lao and Myanmar (Marcombe et al 2019; Kawada et al 2010). The negative genetic association between the 1016 and 1534 kdr indicate that the two genes are not independent from each other. How did the authors have addressed this issue in the analysis?

>> Actually, there is only one gene and the 1016 and 1534 sites are linked. What happens with Asian haplotypes is that mutations in 1016 and 1534 evolved independently. Therefore, one may find an individual *kdr* homozygotes at 1016 but not at 1534 site. In the American haplotypes, it is likely that the *kdr* at 1016 evolved from a haplotype with *kdr* in 1534. Therefore, we find homozygous *kdr* at 1534 with either 1016 variant (V or I). Homozygous *kdr* at 1016 and not at 1534 are rarely reported. In our experience, whenever this combination appears to exist, further work shows it was due to a methodological problem.

Knowing that the authors have mosquito samples from multiple origin (regions) and time scale, would it be possible to determine where and when the 1534C mutation firstly emerged  (eg Africa, Latin America, Asia?)
I wonder why the authors didn’t combine NGS plus classical population genetics tools to determine the possible “routes” of resistance spread ? Please justify.

>> Very good point. That is our dream! The Yale lab has a rich collection with samples from diverse regions. Population genetic studies were published by the Yale team with those samples, highlighting phylogeographic patterns among *Ae. aegypti* populations worldwide. In the case of this study however we were dealing with fragments of a very conserved gene, that is in also subjected to purifying selection. Although we could indeed corroborate the presence of haplotypes unique to Asia and to America/Africa , we did not observe enough diversity in these loci to investigate the genetic origin and population structure worldwide.

Reviewer #3: My only suggestion to improve the presentation of this research is to include a table that shows a summary of the number of individuals use per population or at least per country. That information can be currently obtained by adding samples in the tables provided. A summary table will be useful to gather this information at a glance

>> Agreed. This information was added in Supplementary Table 1

Figure Files:
While revising your submission, please upload your figure files to the Preflight Analysis and Conversion Engine (PACE) digital diagnostic tool, <https://pacev2.apexcovantage.com>. PACE helps ensure that figures meet PLOS requirements. To use PACE, you must first register as a user. Then, login and navigate to the UPLOAD tab, where you will find detailed instructions on how to use the tool. If you encounter any issues or have any questions when using PACE, please email us at [figures@plos.org](mailto:figures@plos.org).
>> Figures were submitted to PACE, which corrected Figures 2, 3 and 5. These new versions were submitted

Data Requirements:
Please note that, as a condition of publication, PLOS' data policy requires that you make available all data used to draw the conclusions outlined in your manuscript. Data must be deposited in an appropriate repository, included within the body of the manuscript, or uploaded as supporting information. This includes all numerical values that were used to generate graphs, histograms etc.. For an example see here: <http://www.plosbiology.org/article/info%3Adoi%2F10.1371%2Fjournal.pbio.1001908#s5>.
>> The sequences of haplotypes were submitted to GenBank as well as sequencing raw data, which were deposited at Sequence Read Archive available at www.ncbi.nlm.nih.gov/sra (SRA Accession number: PRJNA579141, Biosample number: SAMN13092781), as informed in the body of the text. We also made available to the reader 11 files containing supplementary material.

Reproducibility:
To enhance the reproducibility of your results, PLOS recommends that you deposit laboratory protocols in [protocols.io](http://protocols.io), where a protocol can be assigned its own identifier (DOI) such that it can be cited independently in the future. For instructions see <http://journals.plos.org/plosntds/s/submission-guidelines#loc-materials-and-methods>

>> We have not done this yet, but will provide the protocols soon.
